# Differentiation of Cystic Fibrosis-Related Pathogens by Volatile Organic Compound Analysis with Secondary Electrospray Ionization Mass Spectrometry

**DOI:** 10.3390/metabo11110773

**Published:** 2021-11-11

**Authors:** Jérôme Kaeslin, Srdjan Micic, Ronja Weber, Simona Müller, Nathan Perkins, Christoph Berger, Renato Zenobi, Tobias Bruderer, Alexander Moeller

**Affiliations:** 1 Department of Chemistry and Applied Biosciences, Swiss Federal Institute of Technology, Vladimir-Prelog Weg 1-5/10, 8093 Zurich, Switzerland; jerome.kaeslin@org.chem.ethz.ch (J.K.); simona.o.mueller@gmail.com (S.M.); zenobi@org.chem.ethz.ch (R.Z.); 2 Division of Respiratory Medicine and Childhood Research Center, University Children’s Hospital Zurich, Steinwiesstrasse 75, 8032 Zurich, Switzerland; srdjan.micic@kispi.uzh.ch (S.M.); Ronja.Weber@kispi.uzh.ch (R.W.); 3 Clinical Chemistry and Biochemistry, University Children’s Hospital Zurich, Steinwiesstrasse 75, 8032 Zurich, Switzerland; nathan.perkins@kispi.uzh.ch; 4 Division of Infectious Diseases and Hospital Epidemiology, University Children’s Hospital Zurich, Steinwiesstrasse 75, 8032 Zurich, Switzerland; christoph.berger@kispi.uzh.ch; 5 Department of Chemistry and Industrial Chemistry, University of Pisa, Via Giuseppe Moruzzi, 13, 56124 Pisa, Italy

**Keywords:** cystic fibrosis, pathogen profiles, secondary electrospray ionization, high-resolution mass spectrometry, recursive feature elimination, putative compound identification, volatile organic compounds

## Abstract

Identifying and differentiating bacteria based on their emitted volatile organic compounds (VOCs) opens vast opportunities for rapid diagnostics. Secondary electrospray ionization high-resolution mass spectrometry (SESI-HRMS) is an ideal technique for VOC-biomarker discovery because of its speed, sensitivity towards polar molecules and compound characterization possibilities. Here, an in vitro SESI-HRMS workflow to find biomarkers for cystic fibrosis (CF)-related pathogens *P. aeruginosa*, *S. pneumoniae*, *S. aureus*, *H. influenzae*, *E. coli* and *S. maltophilia* is described. From 180 headspace samples, the six pathogens are distinguishable in the first three principal components and predictive analysis with a support vector machine algorithm using leave-one-out cross-validation exhibited perfect accuracy scores for the differentiation between the groups. Additionally, 94 distinctive features were found by recursive feature elimination and further characterized by SESI-MS/MS, which yielded 33 putatively identified biomarkers. In conclusion, the six pathogens can be distinguished in vitro based on their VOC profiles as well as the herein reported putative biomarkers. In the future, these putative biomarkers might be helpful for pathogen detection in vivo based on breath samples from patients with CF.

## 1. Introduction

Confirming the presence and identity of pathogenic bacteria is of key importance for the diagnosis of bacterial infections. Classical diagnostic methods are slow because they involve time-consuming steps such as cultivation followed by biochemical, serological or genetic analysis [1]. Alternatively, bacteria can be identified and specified indirectly by detecting emitted metabolic volatile organic compounds (VOCs) [2]. If some VOCs are characteristic for one particular bacterium, they can be used as markers to indicate the presence of the species in the sample under investigation, e.g., in vitro cultures, blood, urine, saliva, sputum or breath. Using more rapid diagnostic methods to detect pathogen-specific biomarkers allows more timely treatment decisions, allows to monitor the progression of a treatment and might be less invasive [3,4,5].

Sensitive detection of low abundant gaseous biomarkers is typically achieved by gas chromatography–mass spectrometry (GC-MS) [6], electronic nose sensing [7], ion mobility spectrometry (IMS) [8], proton transfer reaction–mass spectrometry (PTR-MS) [9], selected ion flow tube–mass spectrometry (SIFT-MS) [10] or secondary electrospray ionization–mass spectrometry (SESI-MS). As numerous bacterial VOCs bear polar functionalities [11], SESI-MS is a good choice as a detection technique due to its outstanding sensitivity for polar analytes [12] and because it can easily be combined with high resolution MS (HRMS) [13].

Therefore, SESI-MS has demonstrated its strength to detect and differentiate bacteria in preceding research. Prior publications include differentiation of infectious pathogens in vitro [14,15] identification of lung infections and bacterial differentiation in vitro and in mice breath [16,17], differentiation of an antibiotic-susceptible and -resistant bacteria strains in vitro [18], differentiation of oral bacteria in vitro and in human saliva [19], monitoring the time course of a bacterial lung infection in mice breath [20], identification and differentiation of pathogens in contaminated food [21] and monitoring metabolic changes of gut bacteria upon perturbation [22,23]. However, these studies were conducted either in a targeted manner looking only at specific VOCs [18,22,23] or in an untargeted manner by looking only at m/z-features without further investigating the metabolite’s identities beyond a mass-to-charge ratio or a molecular formula [14,15,16,17,19,20,21].

Thus, the aim of this study is to find volatile biomarkers which allow to distinguish between in vitro bacterial cultures and assign putative molecular structures to the features in a systematic approach using SESI-HRMS and MS/MS data in combination with a recently developed annotation tool [24]. The assignment of tentative structure helps for feature comparison across different analytical techniques and aids to formulate hypothetical biomarkers, which can be subsequently confirmed by additional methods such as analysis of gas condensate with liquid chromatography–MS (LC-MS) [25]. We demonstrate this by searching for characteristic biomarkers with discriminative power to differentiate between *Pseudomonas aeruginosa*, *Streptococcus pneumoniae*, *Staphylococcus aureus*, *Haemophilus influenzae*, *Escherichia coli* and *Stenotrophomonas maltophilia*. These six pathogens are frequently associated with lung infections in patients suffering from cystic fibrosis (CF) [26], and prior breath analysis studies with SESI-MS showed that a *Stenotrophomonas maltophilia* lung colonization can be detected [27]. To achieve a successful eradication of the pathogens, early detection of the bacteria and a technique to monitor the progression of medical treatment are highly desirable.

## 2. Results

### 2.1. Pathogen Separation and Predictive Analysis

We have analyzed 30 biological replicates of six pathogen families with SESI-HRMS. The same dilution medium was used for all of the six pathogen strains to allow better comparison of the differences between pathogens excluding the medium or its interaction with the pathogens. As has been pointed out by Rees et al. [28] and Schulz-Bohm et al. [29], previous studies have shown that the produced volatiles are highly dependent on the selected media [30,31,32,33].

**Figure 1:**
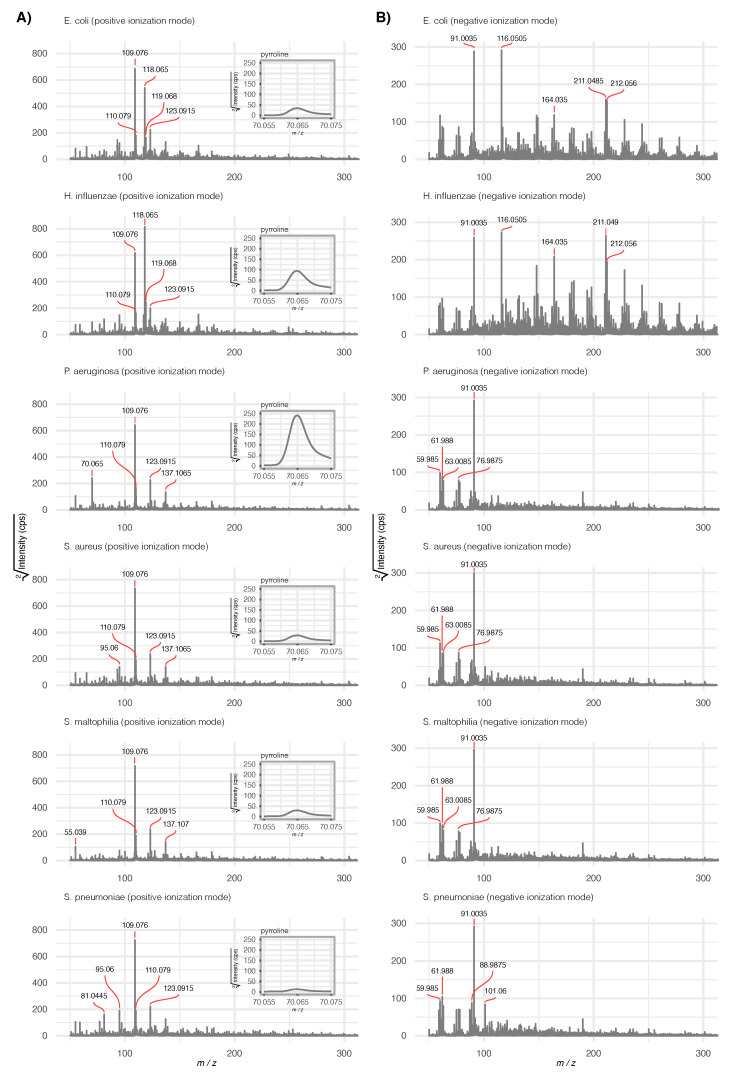
Average mass spectra (averaged over scans and repetitions) for the six pathogens in positive (**A**) and negative (**B**) ionization mode. For better visibility, the intensity scale is square root transformed and only the 50–300 m/z range is depicted. The five most intense signals are labeled. As an example of a characteristic feature for *P. aeruginosa*, a zoomed region corresponding to pyridine ${\left[M+H\right]}^{+}$ is shown in positive mode.

Prior to any further analysis, average raw mass spectra for each pathogen (averaged over scans and repetitions) were calculated for a visual assessment (see Figure 1). While still at an early stage of data analysis, a visible difference between the Gram-negative (*E. coli*, *H. influenzae*) and Gram-positive bacteria (*P. aeruginosa*, *S. aureus*, *S. maltophilia* and *S. pneumoniae*) could be observed. Additionally, pyrolline (m/z value 70.065, positive ionization mode), a confirmed volatile marker for *P. aeruginosa* [34], was handpicked to compare its absolute intensity across all six pathogens (Figure 1A).

**Figure 2:**
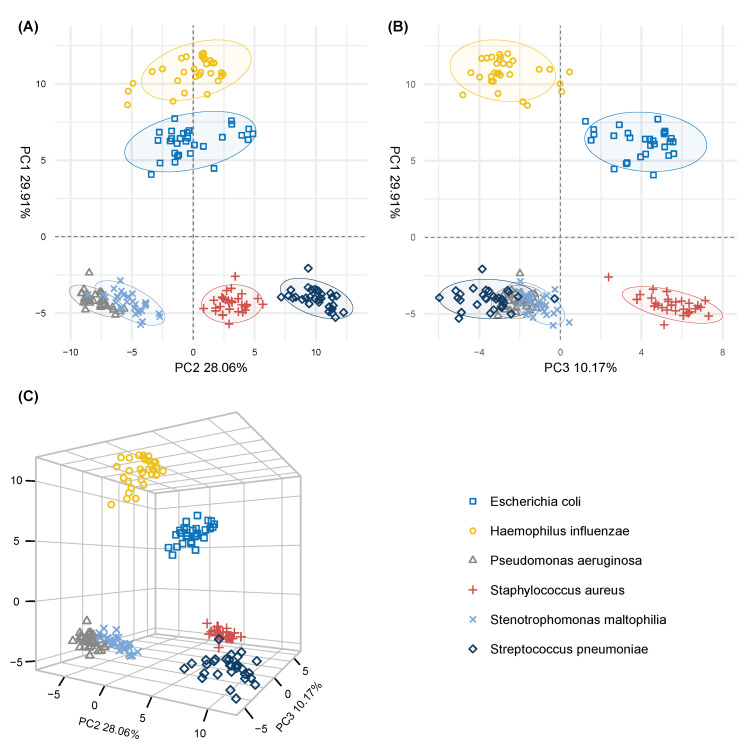
Principal component scores plots of the 6 pathogen groups generated by the extracted 128 m/z-representants. (**A**) PC scores plot of PC1 (29.91%) and PC2 (28.06%), (**B**) PC scores plot of PC1 and PC3 (10.17%) and (**C**) three-dimensional representation of the first three principal components scores. Squares: *E. coli*, circles: *H. influenzae*, triangles (point up): *P. aeruginosa*: plus signs: *S. aureus*, cross marks: *S. maltophilia*, diamonds: *S. pneumoniae*. Colouring was added based on pathogen labels; 90% data ellipses were added (**A**,**B**) for better visual depiction.

The preprocessing of the raw mass spectra (Section 4.3) revealed 939 m/z-features across all samples of which 684 were found to be significantly different (Benjamin–Hochberg-adjusted p<0.05) in pathogen cultures than in sterile medium. By clustering highly correlated m/z-features (Section 4.4), 128 m/z-representants were obtained and subsequently used for principal components analysis (PCA) (Figure 2). The first three principal components (PC) were found to account for almost 70% of total variance in the data which is considered to be a good result. The scores plot of the first two PCs (57.97% of total variance in the data) showed a strong separation between three groups of cultures: *P. aeruginosa* and *S. maltophilia* in one group, *E. coli* and *H. influenzae* in the second group, and *S. aureus* and *S. pneumoniae* in the third group. On the level of single principal components, the separation of closely related *E. coli* and *H. influenzae* from the other four strains was observed along PC1, which accounted for 29.91% of the variance in the data. PC2 (28.12%) captured the differences between *P. aeruginosa* and *S. maltophilia* on one side and *S. pneumoniae* on the other, while PC3 (10.17%) was found to discriminate *S. aureus* and *E. coli* from the other four strains (Figure 2B). The other principal components (not shown here) did not provide any further differentiation between the bacterial species.

After exhibiting that the low-dimensional representation of the data with principal components showed a qualitative trend in the differentiation between the cultures, we assessed the quantitative ability of pathogen profiles to classify the samples into different pathogen groups by supervised machine learning (Section 4.4). In each cross-validation loop of the leave-one-out cross-validation (LOOCV), one sample file was left out and assigned no label. The remaining 179 samples were used to generate pathogen profiles of m/z-representants and subsequently train a classifier with a support vector machine algorithm (SVM). The prediction was then made on the left out sample and compared with the original label. By calculating the cumulative error rate, we recorded an accuracy of 100% for the discrimination between the pathogen groups, i.e., there were no instances of misclassification in any of the 180 predictions in LOOCV. Of note, we have also assessed the classification accuracy by replacing SVM with random forests algorithm [35] trained with 500 decision trees as well as with one-vs-one SVM [36]. We found no changes in accuracy except in the case of random forests algorithm (99.44% accuracy) where one single *S. maltophilia* sample was wrongly classified as *P. aeruginosa*.

### 2.2. Volatile Compounds Associated with Pathogen Strains

As we demonstrated that the overall pathogen profiles can be used to classify samples into different pathogen groups, we sought to identify the subset of potential compounds that was the most informative for a single pathogen group when compared to the other five. We focused on the compounds with higher abundance in the pathogen group in question, because the work presented here is part of a larger project to detect volatile infection markers in breath. Compounds with higher intensities will be more likely detected in breath, where the concentrations will likely be lower than the volatiles detected from headspace from single bacteria cultures. In addition, focusing on the markers with higher intensity for the respective pathogen versus the other five minimizes the likelihood that the characteristic volatile marker is simply originating from a stronger consumption of media compared to the other pathogen families. The inverse case, that all other five pathogen families consume the media more than the single pathogen, which would also result in a relative higher intensity for the pathogen group versus the other groups is assumed not to be likely, with the investigated set of pathogens from different pathogen strains.

The ranking of the m/z-representants according to their discriminatory strength was established for each pathogen group separately with SVM-recursive feature elimination (SVM-RFE) [37]. The pathogen group in question was compared in a two-class model to the other five labeled as a single new group. In this way, six different rankings were produced. Given the ranks, the top 10% of the m/z-representants (rounded down to 12 from 10% of 128) were selected per pathogen group. The 12 m/z-representants associated with the corresponding pathogen group were further reduced by selecting only those m/z-representants with a higher intensity expression in the pathogen group in question when compared to each of the other five. For that, five one-tailed Mann–Whitney U-tests were performed per m/z-representant with Hochberg-adjusted [38] *p*-value threshold set to 0.05. As a result we isolated in total 31 m/z-representants: six for *E. Coli*, four for *H. influenzae*, four for *P. aeruginosa*, five for *S. aureus*, three for *S. maltophilia* and nine for *S. pneumoniae*.

**Figure 3:**
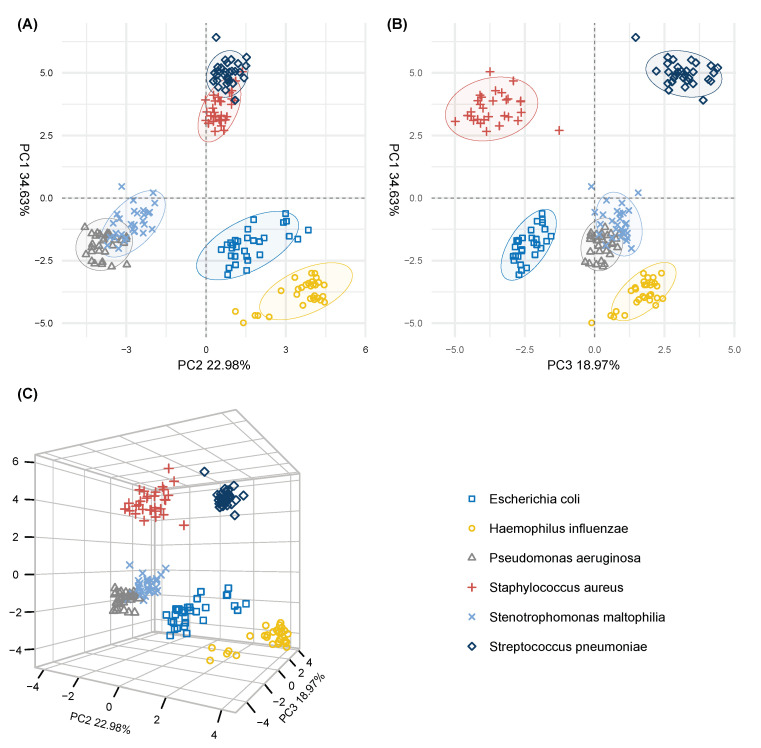
Principal component scores plots of the 6 pathogen groups generated by the selected 31 m/z-representants. (**A**) PC scores plot of the first two principal components (58.49%). (**B**) PC scores plot of the first and third principal component (52.84%). (**C**) three-dimensional representation of the first three principal components scores. Same coloring and data ellipses were added as in Figure 2.

From the selected 31 m/z-representants, we generated principal component scores plots to visualize differences between the pathogens and compare the plots with the broader case of 128 m/z-representants (Figure 2). The first three principal components (Figure 3) accounted for approximately 77% of the variance in the data and showed a visible tendency in separating the pathogen groups. When compared to the PC plots of the 128 representants, we found that the adjacent pathogen clusters in Figure 2A (first two dimensions) showed similar proximity when 31 m/z-representants were used (Figure 3A). Interestingly, the closeness of *P. aeruginosa* and *S. maltophilia* clusters was evident in both cases, indicating that both pathogens were more alike in their metabolite profiles even if the more discriminative variables were selected. However, a noticeable difference was found in the separation between Gram-positive bacteria (*S. aureus* and *S. pneumoniae*) and Gram-negative bacteria (*E. coli*, *H. influenzae*, *P. aeruginosa* and *S. maltophilia*), where the difference was fully captured by the first principal component (34.63%). For the sake of consistency, we also assessed the classification accuracy with SVM by adding the rigorous selection process above when building training data sets in LOOCV. We achieved the same average accuracy score (100%) as in Section 2.1 where full pathogen profiles were used.

The compound identification work was done for all m/z-features contained in the clusters given by the m/z-representants. One cluster (associated with *S. pneumoniae*), encompassing 24 features of which 15 had a mass defect 0.4<Δm/z<1, was excluded at this stage for further analysis because of the unfeasible high number of features and the unlikeliness of metabolites exhibiting such characteristic m/z values [39]. The remaining 30 m/z-representants contained 94 m/z-features, from which for 33 it was possible to assign likely compound structure based on the available MS^2^ data. The most likely compounds were proposed by a literature comparison to assign them to three groups: First, known volatile markers for the investigated pathogen or another bacteria; second, known microbial metabolites; and third reported metabolites with either an entry into the Human Metabolome Database (HMDB) or Kyoto Encyclopedia of Genes and Genomes (KEGG) database (Section 4.5). In summary, we could putatively identify 33 compounds, the elemental composition could be assigned to 47 with the available MS^2^ data and 14 m/z-features were listed with their m/z values. For the complete list containing m/z values, molecular formulas or compound names, we refer the reader to the Table S5.

For *P. aeruginosa*, we could putatively identify the following compounds: pyrroline, pyrrole, 2-methylbutanenitrile and nicotinic acid. Pyrroline (C15668) was reported as a volatile marker for *P. aeruginosa* measured with an ambient MS (Hu et al. [34]). Pyrrole (C19907) was reported as a volatile marker for *P. aeruginosa* measured with GC-MS (Filipiak et al. [40]). Interestingly, both of the compounds have been reported as being present with high intensities during the early linear growth phase with culture experiments. 2-Methylbutanenitrile (C21525), as well as pyrroline and pyrrole, have been reported by Bean et al. [41] who investigated 24 different clinical isolates and putatively identified these three compounds with GC×GC-TOF. Nicotinic acid (C00253) is a novel putative volatile marker for *P. aeruginosa*, which is also known as vitamin B3. It has been reported as a microbial marker but not as a volatile marker for *P. aerugionas* (KEGG, map01120). A metabolite of nicotinic acid, methyl nicotinate has been reported as a volatile marker for *Mycobacterium tuberculosis* (Sethi et al. [3]). Although the boiling point of nicotinic acid is higher than methyl nicotinate (boiling point: $292.5\pm 13{\;}^{\circ }C$ vs. $209.0\pm 0{\;}^{\circ }C$), it is still in the range of the detection capabilities of SESI-HRMS for semi-volatile compounds (see Section 4.5).

The following four novel volatile markers for *S. maltophilia* could be putatively identified: benzothiazole, threo-(homo)2-isocitrate, 2-propionyl-1-pyrroline and troponine. Benzothiazole (HMDB32930) has already been reported as a volatile released from Bacillus strains but not from *S. maltophilia* (Chao-Nan He et al. [42]). Threo-(homo)-2-isocitrate (C16597) has an entry in KEGG as microbial metabolite (map01120). 2-Propionyl-1-pyrroline (HMDB34883) has not been reported as a microbial metabolite, but is closely related to 2-acetyl-1-pyrroline which is produced by a range of bacteria (Routray and Rayaguru [43]). Tropinone (C00783) has not been reported as microbial metabolites.

For *H. influenzae*, *N*-(3’-methylthio)propylmalic acid isomers (C17215, C17214) and aminonitrophenol isomers (C19321, C19322, C19323) are proposed as novel volatile markers, which have not yet been reported as microbial metabolites.

For *E. coli*, phenylacetylglycine and *N*-ethylphenyl-acetamide could be putatively identified. Phenylacetylglycine (C05598) has been reported as gut microbiota metabolite (Yap et al. [44]). *N*-ethylphenyl-acetamide (C11487) has not been reported as microbial metabolites.

For *S. aureus*, 2-hydroxy-2,4-hexadienoic acid and the *m*- and *p*-benzenediol isomers could be putatively identified. We report 2-hydroxy-2,4-hexadienoic acid (C11354) and the the *m*- and *p*-benzenediol isomers (C01751, C00530) as putative novel markers, both of which have been reported as microbial metabolites (KEGG, map01120). Diethanolamine (C11260) has not been reported as a microbial metabolite.

*S. pneumoniae* had the highest number of characteristic features among this pathogen group, of which 18 were putatively assigned. Pyrimidine (C00396) has previously been reported as an elevated volatile marker for *S. aureus* and *S. Typhimurium* with SESI-MS (Zhu et al. [14]), and 2-acetylthiazole (HMDB0032964) was reported as volatile marker for Bacillus strains with gas chromatography mass spectrometry (Li et al. [45]). Benzylideneacetone (HMDB0031617) has been reported for bacteria *Xenorhabdus nematophila* (Ji et al. [46]) and t-muurolol (C20184) as microbial metabolite for a marine *Streptomyces* sp. (Ding et al. [47]), while 2-methylpropanoic acid (C02632), 2-furanmethanol (C20441) styrene- cis-2,3-dihydrodiol (C07084) are listed in KEEG as microbial metaboiltes (map01120). The remaining eleven putatively identified compounds have so far not been reported as microbial metabolites: 1,2- or 1,3-propanediol isomers (isomeric C00583/ C02457), octanoic acid (C06423), 2,3-dihydroxy-3-methylpentanoate (C06007), 2-propanoylthiazole (HMDB37168), beta-spathulene (HMDB36416), farnesal (C03461), imidazole- or pyrazole-4-methanol isomers (isomeric HMDB60768/C05562), 2- or 3-acetylthiophene isomers (isomeric HMDB33133/HMDB33134), 2-acetyl-3-methylpyrazine (HMDB30001), 9-oxononanoic acid (C16322) and undecenoic acid (C13910).

**Figure 4:**
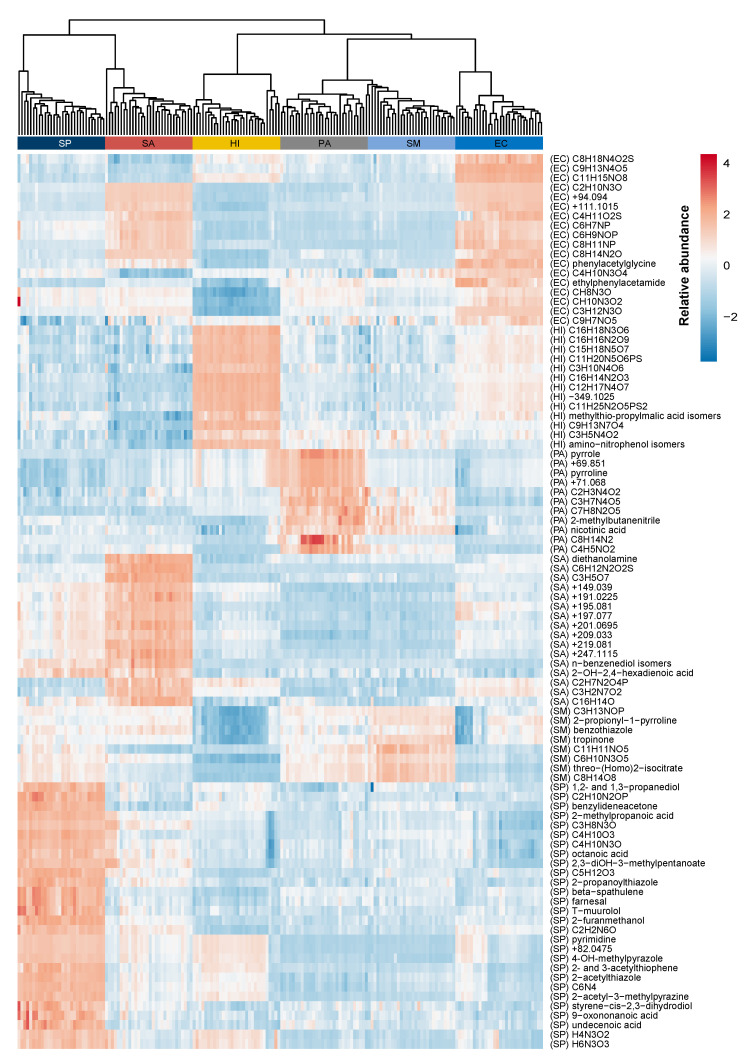
Heatmap of the 94 selected m/z-features across all 6 pathogen groups. Cell colors (range from red (high relative intensity) to blue (low relative intensity)) correspond to relative compound intensity (mean centered and divided by the standard deviation). The top dendrogram (Euclidean distance, average linkage method) shows the relationship between pathogen samples. EC: *E. coli*, HI: *H. influenzae*, PA: *P. aeruginosa*, SA: *S. aureus*, SM: *S. maltophilia*, SP: *S. pneumoniae*. The putative compound names, elemental compositions and m/z values are shown as row names (see also Table S5). The pathogen of the associated two-class model from which a given compound was selected is provided in the brackets.

After having completed the compound identification work, we decided to visualize the relative intensity differences in a heatmap (Figure 4) of all 94 m/z-features and assess the relationship between the pathogens by means of hierarchical clustering analysis (HCA, Euclidean distance measure, average linkage method [48]). The dendrogram in Figure 4 shows, as is also visible in the PC scores plots (Figure 3) using m/z-representants, that *P. aeruginosa* and *S. maltophilia* are very similar to one another as they cluster early as the function of the cluster tree height. When looking at the selected features a group of nitrogen containing chemicals, including pyrrole, pyrroline, 2-methylbutanenitrile and nicotinate showed a higher relative abundance in *P. aeruginosa* than in *S. maltophilia*, while a group of highly oxidized compounds O_5_-O_8_, including the putatively identified threo-(homo)-2-isocitrate showed higher intensities for *S. maltophilia* than for *P. aeruginosa*. The Gram-positive bacteria *S. pneumoniae* and *S. aureus* tend to cluster together and are farthest away from the four Gram-negative bacteria. This was also confirmed in Figure 3. When compared to each other, *S. pneumoniae* showed a large group of compounds with higher relative intensity differentiating it from *S. aureus*. This group was not particularly highly oxygenated and it was possible to putatively identify 18 of the 28 m/z-features in this group. For *S. aureus*, higher relative intensities were observed for 16 m/z-features, including diethanolamine, *n*- and *p*-benzenediol and 2-hydroxy-2,4-hexadienoic acid. Most of the remaining m/z-features of this group could not be annotated with a molecular composition and are listed as m/z values. Interestingly, the dendrogram in Figure 4 shows that *H. influenzae* is further away from *E. coli* than *E. coli* to *P. aeruginosa* and *S. maltophilia*. This is somehow less obvious from the PC scores plot in Figure 3. For *H. influenzae*, a group of nitrogen-containing compounds, mostly highly oxygenated O_3_-O_9_ markers, including amino-nitrophenol isomers, showed higher abundance for *H. influenzae* than for *E. coli* but were also present in *E. coli*. The only marker without nitrogen in this group was (3’-methylthio)-propylmalic acid. On the other side, another group of nitrogen-containing compounds with only few highly oxygenated markers showed higher relative intensities for *E. coli* than *H. influenzae*, including phenylacetylglycine and *N*-ethylphenyl-acetamide. These compounds had a significantly lower relative intensity in *H. influenzae* even when compared with the other pathogens.

## 3. Discussion

The aim of this work was to determine if the six CF-associated pulmonary pathogens can be distinguished with SESI-HRMS and to isolate and identify the most informative VOCs. This work lays the groundwork to provide a basis for the detection of infections in breath of people with CF. One of the main strengths of this multiple pathogen study was its high number of biological replicates (n=180 biological samples with 30 biological replicates for each of the six pathogens). Another advantage when compared to most other studies (see, e.g., in [14,28,49,50]) was the usage of SESI-HRMS instead of GC-MS. This allowed for inclusion of positively as well as negatively charged ions, which are rarely measured with GC, resulting in rich VOC profiles of the pathogen groups. To the best of our knowledge, this is the first time that the selected six CF-associated pathogens were investigated with SESI-HRMS.

The extracted VOC profiles were complex, as a large number of m/z-features were acquired from the measurements of the samples. By applying dimensionality reduction based on the Pearson correlation coefficients between the features by means of a static tree cut in the hierarchical tree, we intended to group isotopologues, adducts, fragments and metabolically linked compounds into clusters. As SESI-HRMS is an on-line method not relying on pre-separation, all these species are detected together—no matter whether they are from the same compound (isotopologues, adducts, fragments) or from their biological relationship in a metabolic pathway. If the latter is the case, it would be interesting to study the metabolic relationship in more detail in future studies.

This study has demonstrated that the full VOC profiles of the six pathogens allowed a qualitative differentiation of bacterial species by simple PCA. Interestingly, when using only the most informative features from our data pipeline, the differentiation between the Gram-negative and the Gram-positive bacteria was very evident. Another key point in our work was the inclusion of supervised machine learning as a method to assess the predictive power of the pathogen profiles. As a result, we concluded that it was possible to assign single samples to different bacteria strains with a very high accuracy (100%). We argue that this positive result demonstrates the potential application of VOCs as diagnostic markers. Nevertheless, despite using LOOCV to assess the prediction of pathogen groups, an independent validation set would still be needed in order to evaluate the robustness of our statistical procedure. The importance of using supervised models was also indicated in the seminal work of Rees et al. [28] where the authors report the average prediction accuracy as one of their main results. As explained there, the translation of profiles or selected markers found in profiles into clinical settings needs test parameters such as accuracy to be reported in order to evaluate reliability and precision of the differentiation between the pathogens.

When compared to other studies, Nizio et al. [49] is conceptually the most closely related study, with a focus on CF-associated pathogens including *P. aeruginosa*, *S. Maltophilia*, *H. Influenzae*, *S. Pneumoniae* and two others, but not *S. Aureus* or *E. Coli*. Interestingly, Nizio et al. could not differentiate their set of six pathogens relying on GC×GC-TOF measurements and unsupervised analysis with principal components. Notably, a comparison of the results of different multiple pathogen studies is limited because different sets of pathogens have been investigated in each study. Each newly investigated pathogen or non-investigated pathogen can change the final set of characteristic compounds, which is specific only to differentiate the pathogens of the conducted study.

We hypothesized that by recursively eliminating the majority of the variables in a two-class model and comparing one pathogen against the others we could capture the most informative features of the pathogen in question. This, however, can also include features which are overly underexpressed for the considered pathogen or even features which are similarly expressed for more than one pathogen group. The intentional post hoc many-to-one tests were applied to single out only those with a higher relative abundance for the one pathogen group of interest, which is not meant to undervalue other high ranking metabolites. Nevertheless, it would be feasible to repeat the current study where the identified compounds are used as predictive variables.

An effort was made to putatively identify the resulting 94 most informative m/z-features based on available MS^2^ data which resulted in 33 putatively identified compounds and the assignment of 47 molecular formulas. The molecular formula patterns indicate groups of related molecules for different pathogens, such as nitrogen containing compounds for *P. aeruginosa*, and groups of highly oxidized compounds with higher relative abundances for some of the investigated pathogens. It must be pointed out that this is a first explorative study and the compounds have only been putatively identified. The relevant volatiles (e.g., the ones which can also be detected in breath of children with cystic fibrosis and specific infections) will be investigated at a later stage for unequivocal identification with either GC×GC-TOF-MS or liquid chromatography-tandem mass spectrometry analysis (LC-MS/MS) [25]. Furthermore, note that the reported metabolites are characteristic for the herein presented study, i.e., that other features might be found to be characteristic if an alternative medium is used, an alternative medium temperature is set or alternative pathogens are compared against each another. Consequently, there is no guarantee that the same distinctive features would be found in vivo under physiological conditions. We plan to follow up on this work with a thorough compound identification work for in-depth analysis of biological pathways.

The data analysis workflow leading to the above results was motivated by the work of Rees et al. in [28]. Specifically, we incorporated their idea of using the inner mechanics of the supervised machine learning algorithm to rank the metabolites as well as of using supervised machine learning as a way of quantifying the predictive power of the metabolic profiles.

## 4. Materials and Methods

### 4.1. Pathogen Strains and Sample Preparation

Quality control strains of six pathogens were selected for continuous headspace experiments. The selected pathogens from the American Type Culture Collection (ATCC) were *E. coli* (ATCC 25922), *H. influenzae* (ATCC 9006), *P. aeruginosa* (ATCC 27853), *S. aureus* (ATCC 29213), *S.maltophilia* (ATCC 13636) and *S. pneumoniae* (ATCC 49619). BD Chocolate Agar (GC II Agar with IsoVitaleX), ready-to-use-plated media (Becton, No. 254089), was used for *H. influenzae* and BD Columbia Agar with 5% Sheep Blood (Becton, No. 254089) for the other strains, respectively. For each experiment, the quality control strain was first subcultured on Agar plates and incubated at $37{\;}^{\circ }C$ in 5% CO_2_ for 24 h.

Subsequently, 8 mL Brain Heart Infusion (BHI) was inoculated by the sub-cultured quality control strains and incubated at $35{\;}^{\circ }C$ in air for 24 h. A volume of 2 mL of each sample was transferred to the headspace samplers and stored in an incubator at $37{\;}^{\circ }C$ until measurement by SESI-HRMS. In total *n* = 30 biological replicates of each pathogen strain in BHI medium and *n* = 11 sterile media were prepared. Further information about the used pathogen strains and sample preparation materials can be found in the Supplemental Information, Section 1.1.

### 4.2. Continuous Headspace Analysis with SESI-HRMS

**Figure 5:**
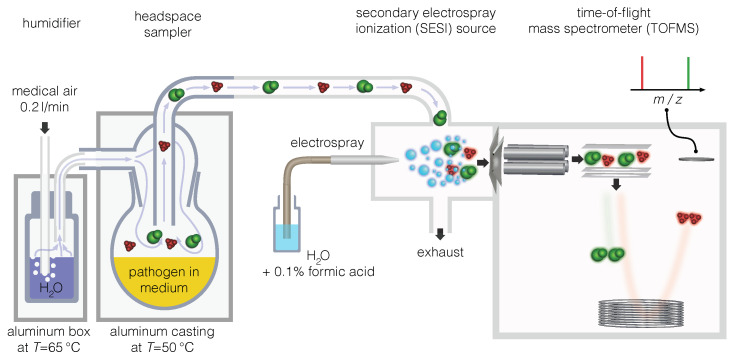
Setup for bacteria continuous headspace analysis with SESI-HRMS.

The setup for continuous headspace analysis of pathogen cultures is shown in Figure 5. A SESI-TOF ion source (Fossiliontech, Madrid, Spain) was connected to a TripleTOF 5600+ mass spectrometer (TTOF 5600+, Sciex, ON, Canada). Mass spectra were acquired in the mass to charge (m/z) range between 50 and 500 with an accumulation time of 1 s. The temperatures of the SESI ion source were set to sampling line ( 130 ${}^{\circ }C$), ion source core ( 130 ${}^{\circ }C$) and nitrogen gas supply ( 130 ${}^{\circ }C$). The effective temperature of the core of the SESI ion source was below the boiling point of water ( 100 ${}^{\circ }C$). Otherwise, the ionspray would have evaporated resulting in no signal. Nanospray capillaries were used with 360 μm outer diameter, 20 μm inner diameter, 50 cm length (TT-360-20-20-N-5, New Objective, Littleton, MA, USA), and were cut to 30 cm length. The TTOF 5600+ settings were ion spray voltage (±4500V), CUR (10), gas 1 (24), gas 2 (24), CE (±10eV) and declustering potential (±20V). The effective ion spray voltage for the SESI-MS was ±3500V.

Samples were analyzed in custom-made headspace samplers, which were designed for minimal background signals with either highly inert glass or PTFE surfaces and tubes. They could readily be disinfected and cleaned by high-purity grade solvents. A stream of 200 mL/min filtered medical air was humidified with a gas-washing bottle and passed through the headspace of the samplers, which were kept at 50 ${}^{\circ }C$ in a custom-made, heated aluminum box throughout the measurements. Further information about the sampling system can be found in the Supplemental Information, Section 1.2.

Biological replicates of each pathogen in BHI media and sterile media were measured by continuous headspace SESI-HRMS analysis. The headspace sampler was detached when the total amount of detected signal (total ion current chromatogram, TIC) reached a signal plateau for a time period of 100 s. On each consecutive measurement day, one headspace sample of each quality control strain was measured during a total of 30 measurement days. Negative and positive ionization modes were successively measured every day, pathogen by pathogen. The order of the measured pathogens was randomized for each day.

### 4.3. Data Preprocessing

Data files acquired from the measurements of pathogen and sterile medium samples were converted to the open .mzXML format using the MSConvert (ProteoWizard version 3.0x, [51]) and further processed in R v3.4.4 (R Foundation for Statistical Computing, Vienna, Austria). Mass spectra were resampled using piecewise cubic Hermite interpolation [52] onto a linearly spaced m/z-axis with a resolution of 0.0005 ($9\times 10^{8}$ data points, 50–500 m/z-axis range). The total ion chromatograms (TICs) of each experiment were calculated by integration and used to distinguish between the mass spectra originating from sample and base signals. For each sample, peak picking was performed on the average mass spectrum calculated over scans generated by the sample signal. Peak positions (m/z-features) were then used to centroid the peaks by integration in each spectrum, yielding intensities of the m/z-features and their time traces per experiment. To exclude the features which do not originate from the sample, only those m/z-features with a positive correlation (Pearson correlation coefficient >0.7) between the feature time trace and the TIC were selected. Additionally, when compared over all samples, m/z-features which were selected in less than 80% of the samples of one sample group (pathogen group or sterile medium) were excluded in order to avoid using inconsistently measured features from further analysis. Normalization of the data was performed by averaging the intensities of the m/z-features during the scans generated by the sample signal and dividing by the averaged TIC over the same scans (i.e., normalization with respect to TIC). The normalized intensities were $\log_{10}$-transformed and arranged into a n×k matrix for further statistical analysis, with *n* the number of samples and *k* the number of m/z-features. For more details on data preprocessing we refer the reader to Supplemental Information, Section 1.3.

### 4.4. Statistical Analysis

The data analysis workflow was motivated by the seminal work of Rees et al. [28] and adapted to our setting. First, the effect of the sterile medium was reduced by applying the same method as in [28]. More precisely, the Mann–Whitney U test [53] together with Benjamini–Hochberg adjustment [54] for *p*-values was used to select the m/z-features which are significantly different in pathogen groups than in sterile media samples. The adjusted *p*-value threshold was set to 0.05.

Dimensionality reduction was performed of the data matrix representing the 6 pathogen groups by clustering the m/z-features with similar intensity profiles across the pathogen samples. Briefly, Pearson correlation coefficients of each pair of m/z-features were used to construct the dissimilarity matrix for agglomerative hierarchical clustering (distance given by 0.5×(1−c), *c* = Pearson correlation coefficient of a pair of features). The resulting dendrogram (cluster tree) was cut at a fixed height of 0.1 grouping m/z-features with similar intensity profiles into clusters. The first principal component of the standardized (mean centered and divided by the standard deviation) m/z-feature set in each cluster was selected as the representant of the cluster (here referred to as m/z-representant). In case of a single element cluster the feature itself was selected as the representant. The m/z-representants were arranged into a data matrix of pathogen profiles for further analysis.

PCA was conducted on the pathogen profiles for low dimensional visualization of the pathogen samples. HCA with Euclidean distance measure and average linkage method [48] was used to analyze the hierarchical relationship between the samples. Prior to PCA and HCA, variables were mean centered and divided by the standard deviation. The predictive power of the pathogen profiles was assessed in a LOOCV with linear SVM [55] adapted for multi-class problems as described in [56]. In order to avoid feature selection bias and overfitting, all the processing steps needed to derive the m/z-representants data matrix of pathogen profiles were repeated in each loop of the cross-validation, see in [57,58]. The ranking of the m/z-representants according to their discriminatory power was performed for each pathogen group separately in one-against-all fashion by SVM-RFE [37].

The data analysis workflow above is very similar to the one in [28]. The main difference was in the choice of the underlying supervised machine learning algorithm, namely, SVM, and recursive feature elimination with SVM as a ranking criterion. In [28], the authors relied on a different algorithm, namely, the random forest algorithm [35], to predict the pathogen membership and the mean decrease in accuracy in the random forest algorithm to rank their metabolites. We refer the reader to Supplemental Information, Section 1.4, for more details on statistical analysis and how SVM is applied for feature ranking.

### 4.5. Putative Compound Identification with SESI-HRMS^2^

For putative compound identification, pathogen strains were measured on an Orbitrap Q Exactive Plus (Orbitrap QE, Thermo Scientific, Waltham, MA, USA) which offers higher resolving power and the capability of trapping low abundant ions. The same bacteria cultivation conditions, headspace setup and flow settings were used for the compound identification experiments as for the screening experiments. The main Orbitrap settings were $250{\;}^{\circ }$C ion transfer temperature, mass resolving power of 280,000 at m/z 200, 55% RF lens, $5\times 10^{6}$ AGC and 3000 ms maximum trapping time. The selective features from the TTOF 5600+ data were used as m/z target list for Orbitrap MS^1^ and MS^2^ with a m/z 0.4 isolation window. Collision-induced dissociation (CID) was performed with N_2_ as collision gas and with 10, 30 and 55 stepped collision energies.

The Orbitrap data was converted to .mzXML and .mgf files using MSConvert (ProteoWizardversion 3.0x, [51]). Information about the detailed compound identification workflow can be found in the Supplementary Material (Section 1.5). Briefly, first the features were isotope filtered; second, irreproducible ${\mathrm{MS}}^{1}$ features (TTOF 5600+ versus Orbitrap QE) were rejected; third, preventing double counting of the same analyte by searching for in-source CID fragments or characteristic electrospray ionisation (ESI) adducts and losses; and fourth, the MS^2^ spectra were analyzed with SIRIUS (4.4.29) [24,59,60] yielding in molecular formulas and chemical structures for some of the features. The chemical structures with the top MS^2^ scores with an KEGG or HMDB database entry are reported as putative assignments. If the SIRIUS interpretation of the MS^2^ data failed to yield a chemical structure, the found molecular formula was instead processed to find metabolic pathways linking features within a cluster. The results of the identification steps are listed in Table S2.

For most m/z-features, multiple compounds were suggested by SIRIUS with either a KEGG or HMDB database entry. Compound candidates were compared with the literature by (1) KEGG database entry, (2) HMDB database entry and (3) Google search with the keywords ‘compound name’, ‘bacteria’ and ‘volatile’. A match with known volatile markers from bacteria were assigned as most likely compounds, followed by known microbial metabolites. In the case of multiple remaining possibilities, the candidate with top SIRIUS MS^2^ matching score was listed. Multiple candidates were listed in case of more than one candidates from the top score up to a score of +30. As an illustrative example, thirteen candidates were suggested by Sirius for the m/z value +194.0815 with the molecular composition C_10_H_11_NO_3_ for *E. coli*. Of these only, phenylacetylglycine was reported as a microbial metabolite from the gut (Yap et al. [44]) which is suggested as the most likely compound. Further information can be found in the Table S3.

For plausibility control, we investigated the volatility and polarity for the putatively identified compounds. SESI can detect compounds with very high boiling points such as fatty acids up to 15 carbon atoms (pentadecanoic acid, boiling point $330.4\pm 5{\;}^{\circ }C$, [25]); amino acids, e.g., l-pyroglutamic acid ($453.1\pm 38.0{\;}^{\circ }C$, [61]); or even formoterol ($603.2\pm 55.0{\;}^{\circ }C$ [62]). The vapor pressure for these compounds with relatively low volatility will be close to 0, therefore we decided to rely on boiling points as volatility estimates. Furthermore, the experimental boiling points for several of the putative, novel markers presented for this set of 6 pathogens are not known. Therefore, for consistent comparison of volatility among all compounds, the predicted boiling points calculated with ACD (ACD/Labs Percepta PhysChem Module, version 2020, Advanced Chemistry Development, Inc., Toronto, On, Canada) obtained from in [63] (ChemSpider) were used as an estimate for compound volatility throughout this manuscript. Three of the suggested compound structures had very low volatility (boiling point $>500{\;}^{\circ }C$) and were very polar (logD <−4). These compounds were also the ones with very low SIRIUS MS^2^ matching scores <−300. Although boiling points are not a strict exclusion criteria we decided to report only the elemental composition and not putative compound structure for compounds with very low SIRIUS MS^2^ scores <−300 (see Table S4). Finally, we summarized the compounds resulting from the putative identification work in a list containing m/z values, formulas or compound names together with KEGG or HMDB entries (see Table S5).

## 5. Conclusions

In this study, the pathogens *P. aeruginosa*, *S. pneumoniae*, *S. aureus*, *H. influenzae*, *E. coli* and *S. maltophilia* were investigated with SESI-HRMS. We have demonstrated by principal component analysis and with supervised machine learning that the six investigated CF-associated pathogens can be differentiated by SESI-HRMS. Additionally, we have isolated the most informative markers which could potentially be applied in clinical settings for disease detection. As SESI-HRMS is an on-line method, it could be used as a breath diagnostic tool for rapid detection of pulmonary pathogens as a painless, non-invasive alternative to sputum collection or bronchoscopy. This would be particularly advantageous for patients with CF that are unable to produce sputum, such as young children and people undergoing cystic fibrosis transmembrane regulator modulator therapy. We shall therefore continue the investigation of bacterial headspaces and pursue validation of our results in vitro, with the goal of developing this technique further for disease detection in vivo.

## Data Availability

The data presented in this study are available on request from the corresponding authors. The data are not currently publicly available as the data is used with another microbiological study at the hospital.

## Supplementary Materials

The following are available online: https://www.mdpi.com/article/10.3390/metabo11110773/s1. Figure S1: Example of a typical TIC recorded in the positive mode during the experiment measuring *P. aeruginosa*. The *x*-axis represents the scans (1 scan = 1 second) and *y*-axis the standardized (mean centered and divided by the standard deviation) TIC. The scans with the standardized TIC >0 belong to signals generated by the pathogen, Figure S2: Process schema of putative assignmen, Table S1: Alignement Table TripleTOF 5600+, Table S2: Target list with structure, Table S3: Putative markers literature comparison, Table S4: Plausibility control, Table S5: List of putatively identified compounds.

## Abbreviations

The following abbreviations are used in this manuscript:

ATCC	American Type Culture Collection
BHI	Brain Heart Infusion
CF	Cystic fibrosis
CID	Collision-induced dissociation
GC	Gas chromatography
HCA	Hierarchical clustering analysis
HMDB	Human Metabolome Database
HRMS	High-resolution mass spectrometry
KEGG	Kyoto Encyclopedia of Genes and Genome
LC-MS/MS	Liquid chromatography–tandem mass spectrometry analysis
LOOCV	leave-one-out cross-validation
MS	Mass Spectrometry
PC	Principal component
PCA	Principal component analysis
RFE	Recursive feature elimination
SESI	Secondary electrospray ionization
SESI-MS	Secondary electrospray ionization-mass spectrometry
SESI-HRMS	Secondary electrospray ionization-high resolution mass spectrometry
SVM	Support vector machine algorithm
SVM-RFE	Recursive feature elimination with SVM
VOC	Volatile organic compound
TIC	Total ion chromatogram
TOF	Time of flight
