# Supplementary material for: Differentiation of Cystic Fibrosis-Related Pathogens by Volatile Organic Compound Analysis with Secondary Electrospray Ionization Mass Spectrometry
Source: Metabolites. 2021 Nov 11;11(11):773. doi: 10.3390/metabo11110773 (PMC8617967; doi:10.3390/metabo11110773)
Supplement: Supplementary file 1 [file metabolites-11-00773-s001.zip › metabolites-1444134-supplementary -revise/Figures/headspace_setup.pdf]

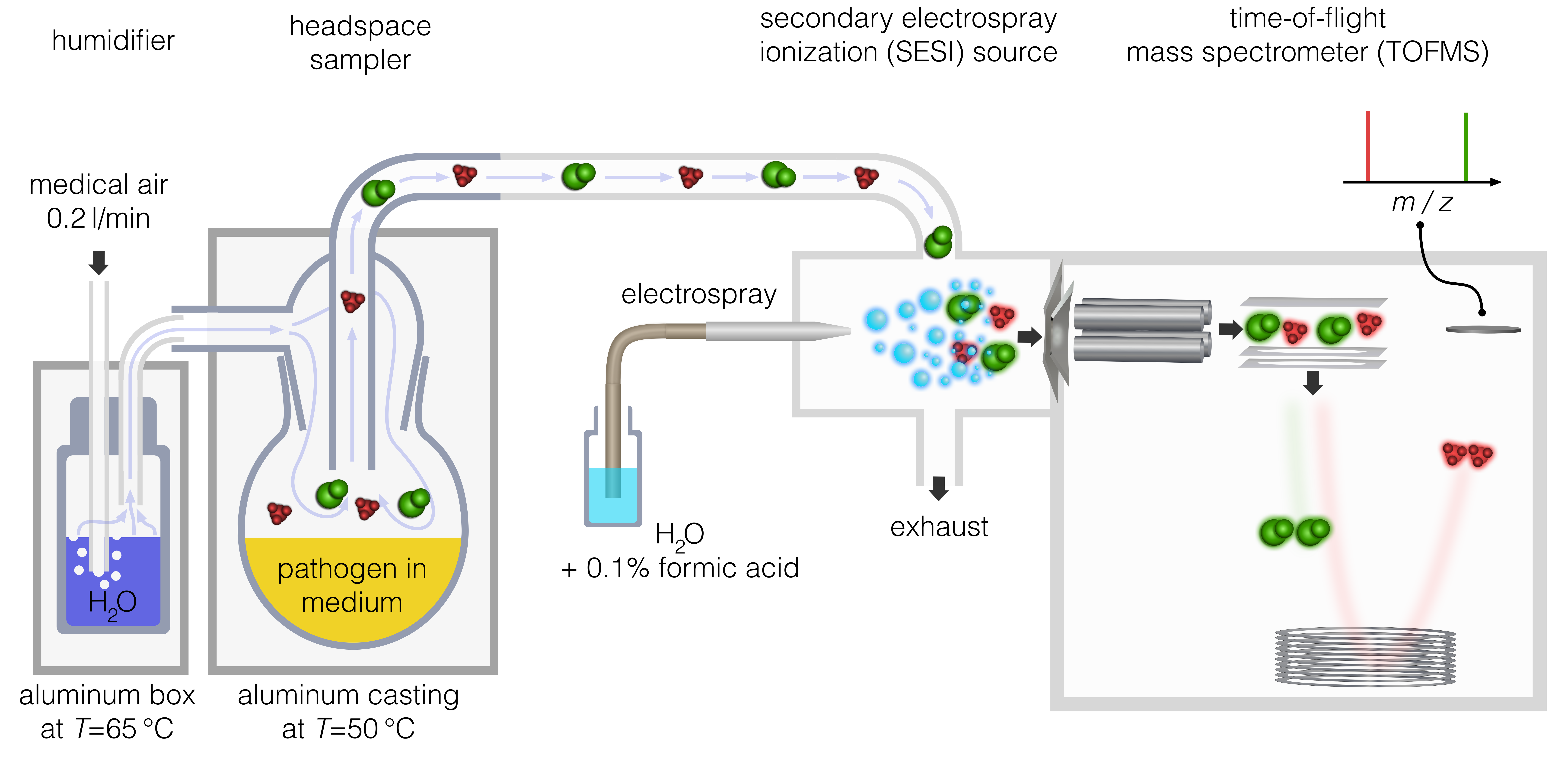
humidifier
headspace sampler
secondary electrospray ionization (SESI) source
time-of-flight mass spectrometer (TOFMS)
medical air 0.2 l/min
m / z
electrospray
H2O
pathogen in medium
H2O + 0.1% formic acid
exhaust
aluminum box at T=65 °C
aluminum casting at T=50 °C
