# Supplementary material for: Differentiation of Cystic Fibrosis-Related Pathogens by Volatile Organic Compound Analysis with Secondary Electrospray Ionization Mass Spectrometry
Source: Metabolites. 2021 Nov 11;11(11):773. doi: 10.3390/metabo11110773 (PMC8617967; doi:10.3390/metabo11110773)
Supplement: Supplementary file 1 [file metabolites-11-00773-s001.zip › metabolites-1444134-supplementary -revise/Figures/pca_pathogen_profiles.pdf]

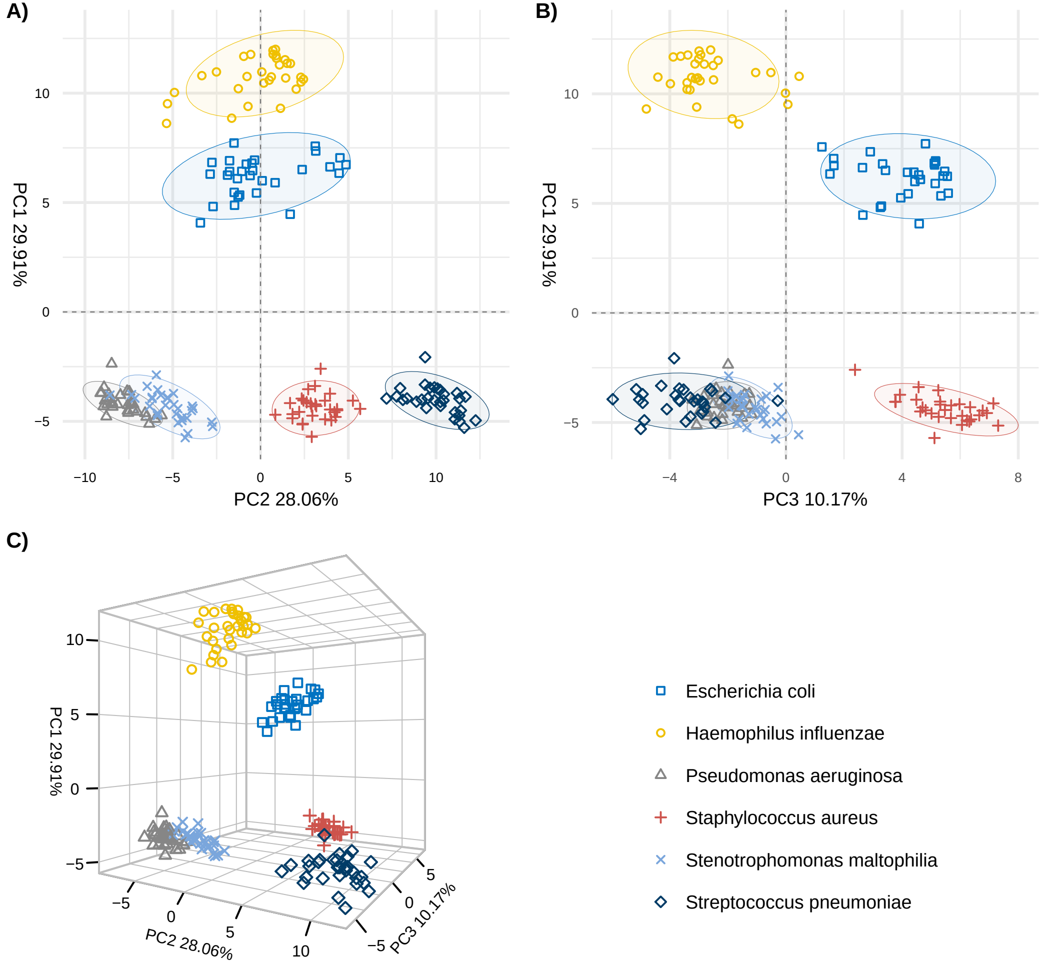
A)
PC1 29.91%
PC2 28.06%
B)
PC1 29.91%
PC3 10.17%
C)
PC1 29.91%
PC2 28.06%
PC3 10.17%
Escherichia coli
Haemophilus influenzae
Pseudomonas aeruginosa
Staphylococcus aureus
Stenotrophomonas maltophilia
Streptococcus pneumoniae
