# Supplementary material for: Differentiation of Cystic Fibrosis-Related Pathogens by Volatile Organic Compound Analysis with Secondary Electrospray Ionization Mass Spectrometry
Source: Metabolites. 2021 Nov 11;11(11):773. doi: 10.3390/metabo11110773 (PMC8617967; doi:10.3390/metabo11110773)
Supplement: Supplementary file 1 [file metabolites-11-00773-s001.zip › metabolites-1444134-supplementary -revise/Figures/pca_pathogen_profiles_selected_top_features.pdf]

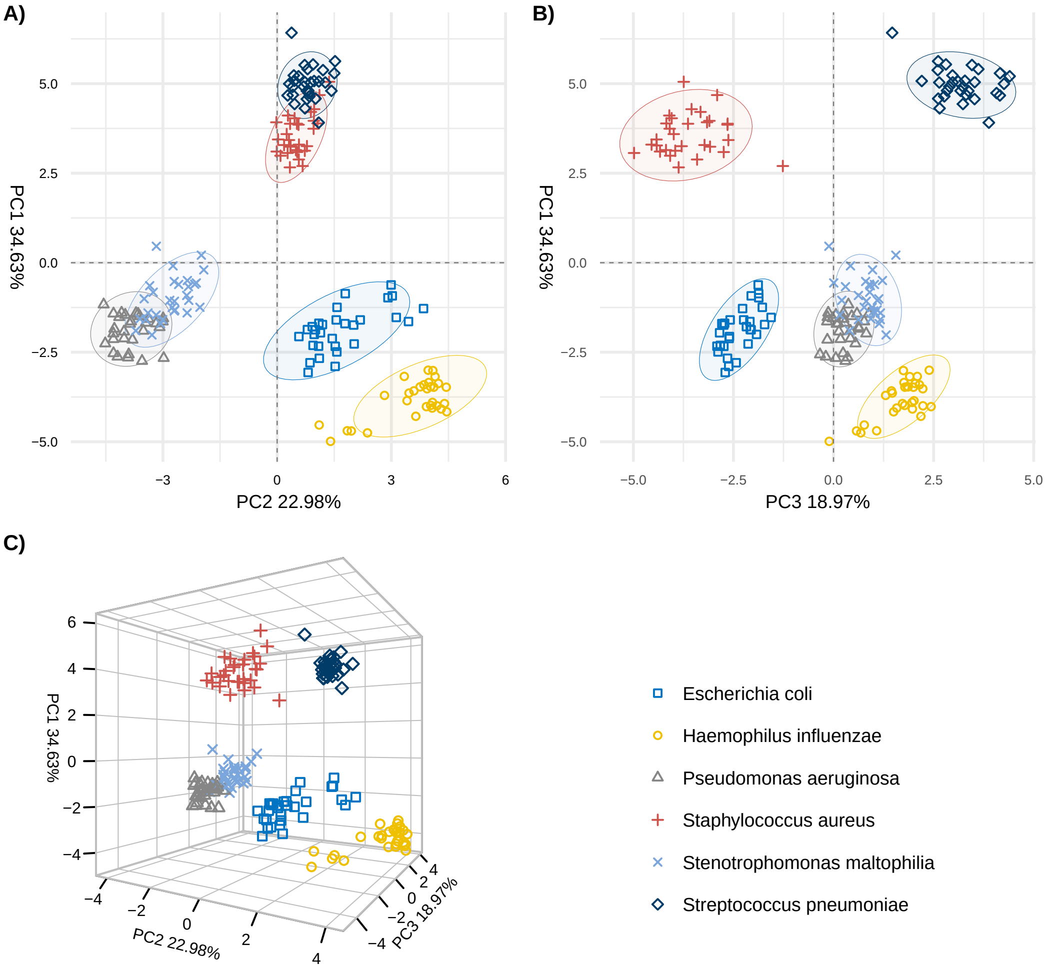

A)
5.0
2.5
0.0
−2.5
−5.0
PC1 34.63%
−3
0
3
6
PC2 22.98%
B)
5.0
2.5
0.0
−2.5
−5.0
PC1 34.63%
−5.0
−2.5
0.0
2.5
5.0
PC3 18.97%
C)
6
4
2
0
−2
−4
PC1 34.63%
−4
−2
0
2
4
PC2 22.98%
4
2
0
−2
−4
PC3 18.97%
Escherichia coli
Haemophilus influenzae
Pseudomonas aeruginosa
Staphylococcus aureus
Stenotrophomonas maltophilia
Streptococcus pneumoniae
