# Supplementary material for: Differentiation of Cystic Fibrosis-Related Pathogens by Volatile Organic Compound Analysis with Secondary Electrospray Ionization Mass Spectrometry
Source: Metabolites. 2021 Nov 11;11(11):773. doi: 10.3390/metabo11110773 (PMC8617967; doi:10.3390/metabo11110773)
Supplement: Supplementary file 1 [file metabolites-11-00773-s001.zip › metabolites-1444134-supplementary -revise/supplemental.pdf]

metabolites

MDPI

Article

Supplemental Information—Differentiation of Cystic Fibrosis Related Pathogens by Volatile Organic Compound Analysis with Secondary Electrospray Ionization Mass Spectrometry

Jérôme Kaeslin [1,†], Srdjan J. Micic [2,†], Simona Müller [1], Nathan Perkins [3], Christoph Berger [4], Renato Zenobi [1], Tobias Bruderer [5,*,‡] and Alexander Moeller [2,6,*,‡]

[1] Department of Chemistry and Applied Biosciences, Swiss Federal Institute of Technology, Vladimir-Prelog Weg 1-5/10, 8093 Zurich, Switzerland
[2] Division of Respiratory Medicine and Childhood Research Center, University Children's Hospital Zurich, Steinwiesstrasse 75, 8032 Zurich, Switzerland
[3] Clinical Chemistry and Biochemistry, University Children's Hospital Zurich, Steinwiesstrasse 75, 8032 Zurich, Switzerland
[4] Division of Infectious Diseases and Hospital Epidemiology, University Children's Hospital Zurich, Steinwiesstrasse 75, 8032 Zurich, Switzerland
[5] Department of Chemistry and Industrial Chemistry, University of Pisa, Via Giuseppe Moruzzi, 13, 56124 Pisa PI, Italy
[6] A list of the members of the Paediatric Exhalomics Group can be found at the end of this article
* Correspondence: tobias.bruderer@dcci.unipi.it (T.B.); Alexander.Moeller@kispi.uzh.ch (A.M.)
† Joint first authors.
‡ To whom correspondence should be addressed.

check for updates

Citation: Lastname, F.; Lastname, F.; Lastname, F. Supplemental Information—Differentiation of Cystic Fibrosis Related Pathogens by Volatile Organic Compound Analysis with Secondary Electrospray Ionization Mass Spectrometry. *Metabolites* **2021**, *1*, 0. https://doi.org/

Received:
Accepted:
Published:

Publisher's Note: MDPI stays neutral with regard to jurisdictional claims in published maps and institutional affiliations.

Copyright: © 2021 by the authors. Licensee MDPI, Basel, Switzerland. This article is an open access article distributed under the terms and conditions of the Creative Commons Attribution (CC BY) license (https://creativecommons.org/licenses/by/4.0/).

1. Materials and methods

1.1. Pathogen strains and sample preparation

The experiments were performed with the following quality control strains from American Type Culture Collection (ATCC): *Escherichia coli* (ATCC 25922), *Haemophilus influenzae* (ATCC 9006), *Pseudomonas aeruginosa* (ATCC 27853), *Staphylococcus aureus* (ATCC 29213), *Stenotrophomonas maltophilia* (ATCC 13636), *Streptococcus pneumoniae* (ATCC 49619). BD Chocolate Agar (GC II Agar with IsoVitaleX), ready-to-use-plated media (Becton, Dickinson and Company, Article number 254089), was used for Haemophilus influenzae (ATCC 9006), for the other quality control strains BD Columbia Agar with 5% Sheep Blood, ready-to-use-plated media (Becton, Dickinson and Company, Article number 254071) was used. BD BBL Brain Heart Infusion, 8 ml (Becton, Dickinson and Company, Article number 220837) was used for sample inoculation.

1.2. Continuous headspace analysis with SESI-HRMS

A calibrated mass flow controller (Bronkhorst AG, model F-201EV-AAD-33-V) was attached at the exhaust of the SESI ion source for monitoring of the flow through the system. Medical respiratory air (PanGas) was filtered with a SGT Click-On Inline Super Clean Gas Purifier (Supelco, Hydrocarbon Trap, 28863-U, Click-On Connectors 1/4″ stainless steel, 28872-U) to remove any possible Hydrocarbon contaminants within the respiratory air. Subsequently, the flow was regulated at 0.2 L/min with a calibrated mass flow controller (Bronkhorst AG, model F-201CV-500-RAD-22-V). The filtered air was humidified with a gas-washing bottle (Quickfit sintered bottle head for use with 125 mL bottles (Merck, article number Z308501-1EA), Quickfit Drechsel bottle (Merck, capacity 125 mL, article number Z308579-1EA), a metal standard taper clip, joint size 24 (Merck, article number Z222380-6EA)) which was 3/4 filled with H_2O (Optima, LC-MS grade, Fisher chemical). The gas-washing bottle was heated to 65 °C using a home-build aluminum heating jacket and kept constant using a Hei-Tec magnetic stirrer with Pt1000 temperature sensor (Heidolph Instruments GmbH & CO.KG, article number 505-30081-00).

Each headspace sampler consisted of a 5 mL round bottom flask (borosilicate glass, NS 14.5/23, Duran), two PTFE valves (Buerkle GmbH, two-way, 4.5 mm outer diameter, 2 mm inner diameter, article number 8610-0010, used for safety reasons), a vacuum receiver (borosilicate glass, cone NS 14.5/23, socket NS 14.5/23, Duran), two Keck Joint Clip (standard taper, joint size 14, yellow), a custom-made PTFE adapter for PTFE tubing (inner diameter 4 mm, outer diameter 6 mm) and vacuum receiver's socket (NS 14.5/23), PTFE tubing (inner diameter 4 mm, outer diameter 6 mm) and PTFE tubing (inner diameter 6 mm, outer diameter 8 mm). The samplers were disinfected with a mixture of 80/20 (v/v, ethanol/water) with LC grade ethanol and cleaned with LC-MS grade methanol.

A custom-made aluminum heating box was fashioned to contain the headspace samplers. A temperature regulator (Hillesheim GmbH, HT55H-10N-2-HAL), a temperature sensor PT100 (Probag AG, article number 84620) and a temperature cartridge (Probag AG, type HS, 6.5 mm diameter, length 100 mm, 230 V, 100 W) were used to keep the temperature of the headspace samplers at 50 °C throughout the entire measurement duration. PTFE tubing was used for all connections. The headspace sampler inlet was attached to the humidifier and the outlet to the inlet of the SESI ion source.

1.3. Data preprocessing

The following section serves as the extended version of the data preprocessing section in the main manuscript.

Raw mass spectra acquired through the experimental measurements of pathogen and sterile medium samples were recorded by Analyst (Version TF 1.7, Applied Biosystems Sciex, Toronto, ON, Canada) in the proprietary WIFF file format. Alignement of the spectra was performed with PeakView software (Version 2.2, Applied Biosystems Sciex, Toronto, ON, Canada) with respect to the exact m/z-values listed in Table S1. The data files were then converted to the open mzXML format using the MSConvert (ProteoWizard version 3.0x, [1]) and further processed in R v3.4.4 (R Foundation for Statistical Computing, Vienna, Austria). All mass spectra were resampled using piecewise cubic Hermite interpolation [2] onto a linearly spaced m/z-axis with a resolution of 0.0005 (9×10^8 data points, 50-500 m/z-axis range). The total ion chromatograms (TICs) of each experiment were calculated by integration and then used to distinguish the mass spectra originating from sample and baseline signals (see Figure S1).

Table S1. Alignement Table TripleTOF 5600+

Positive Ion Mode

Name	Formula	m/z
Acetone	C_3H_6O	+59.04914
Acetone-water	$C_3H_8O_2$	+77.05971
Methyl pyrrolidine	$C_5H_{10}NO$	+100.07569
Dibutylphtalate	$C_{16}H_{22}O_4$	+279.15909

Negative Ion Mode

Name	Formula	m/z
Fatty acid	$C_2H_4O_2$	-59.01385
Fatty acid	$C_{12}H_{24}O_2$	-199.17035
Formic acid dimer	$C_2H_4O_4$	-91.00368
Palmitic acid	$C_{16}H_{32}O_2$	-255.23295
Stearic acid	$C_{18}H_{36}O_2$	-283.26425

For each measured sample the average mass spectrum was calculated over scans generated by the sample signal from which a list of m/z-features was extracted by picking peaks above the absolute intensity of 200 cps (counts per second) in the positive mode and 100 cps in the negative mode. In order to compensate for small variations in the peak positions across the experiments all recorded m/z-features were combined into one

single list from which a kernel density estimate (Gaussian kernel, bandwidth = 0.0025) was computed. Local maxima of the smoothed density function were used to define the final m/z-feature list representative for all samples. The m/z-features were then used to centroid the peaks by integration (mass window $m/z \pm \Delta m/z$, $\Delta m/z = 0.0025$) in each spectrum of each experiment, yielding intensities of the m/z-features and their time traces per experiment. To exclude the features which do not originate from the sample only those m/z-features with Pearson correlation coefficient larger than 0.7 between their time traces and the TIC were selected. Additionally, when compared over all 30 repetitions, m/z-features which were selected in this way in less than 80% of the repetitions of one sample group (pathogen group or sterile medium) were excluded in order to avoid using inconsistently measured features from further analysis. Normalization of the data was performed with respect to the TIC, i.e. by averaging the intensities of m/z-features during the scans generated by the sample signal and dividing by the averaged TIC over the same scans. TIC normalization corrects for the total amount of molecules present in the headspace and thus for the cell number. The relative contribution to the TIC of an individual metabolite is assumed to be constant. It could be possible that commonly expressed metabolites are characteristic for one pathogen because of a difference in their growth rate or viability in the medium. Nonetheless, a non-trivial intensity of a feature in a pathogen still indicates the presence of a particular species. The normalized intensities were $\log_{10}$-transformed and arranged into a $n \times k$ matrix for further statistical analysis, with n the number of samples and k the number of m/z-features.

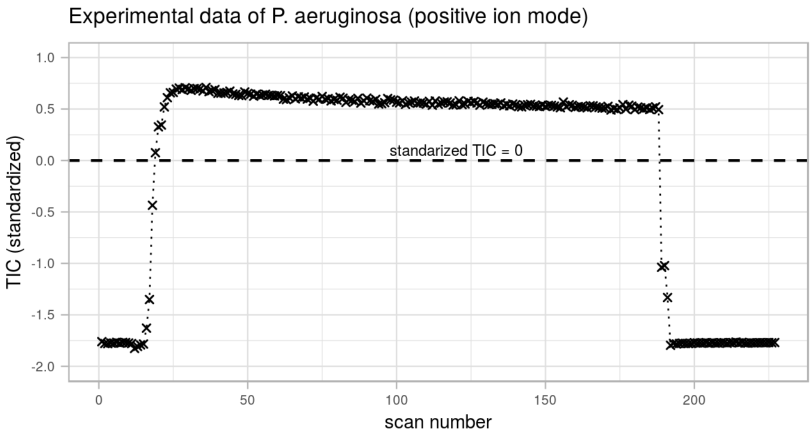

Figure S1. Example of a typical TIC recorded in the positive mode during the experiment measuring *P. aeruginosa*. The x-axis represents the scans (1 scan = 1 second) and y-axis the standardized (mean centered and divided by the standard deviation) TIC. The scans with the standardized TIC > 0 belong to signals generated by the pathogen.

1.4. Statistical analysis

The following section serves as the extended version of the statistical analysis section in the main manuscript. The data analysis pipeline borrows from the ideas in [3]. The main difference was in the choice of the underlying machine learning algorithm, namely support vector machines (SVM) and the ranking procedure of the features by recursive elimination based on SVM. Our choice for the SVM algorithm, instead of a random forest algorithm as employed in [3], is based on the fact that SVM was already successfully applied in prior SESI-HRMS studies [4–7].

As a first step in statistical analysis, the effect of the sterile medium was reduced by applying the same method as in [3]. More precisely, the Mann–Whitney U test [8] together with Benjamini–Hochberg adjustment [9] for p-values was used to select the m/z-features which are significantly different in pathogen groups than in sterile media. The adjusted p-value threshold was set to 0.05. A careful reader will notice that we did not assume that the features were drawn from a normal distribution in each group and therefore applied

a nonparametric test for that reason. Indeed, we conducted the Shapiro-Wilk test for normality [10] on the residuals of each of the m/z-features and found that a majority of the features were not normally distributed. Out of the 939 m/z-features resulting from the data preprocessing (section 1.3) we found that for approximately 80% of the m/z-features the null hypothesis was rejected ($p < 0.05$).

In order to avoid using highly correlated m/z-features simultaneously for further analysis the intensity matrix was reduced by grouping m/z-features with similar intensity profiles. For that purpose, hierarchical clustering with correlation based distance measure was conducted. Pearson correlation coefficients $c_{i,j}$ of each pair of m/z-features i, j were transformed into a distance measure $0.5 \cdot (1 - c_{i,j})$ from which a dissimilarity matrix for hierarchical clustering was constructed. The resulting dendrogram was cut at a fixed height of 0.1 grouping m/z-features with similar intensity profiles into clusters. To represent each cluster with a single element principal component analysis (PCA) was performed on the set of m/z-features of each cluster (intensities of the m/z-features were mean centered and divided by the standard deviation prior to PCA). The major (first) principal component was selected as the representant of the cluster (here referred to as the m/z-representant). In case of a single element cluster the feature itself was selected as the representant. The m/z-representants were arranged into a data matrix of pathogen profiles reducing the dimensionality of the data set.

Principal component analysis (PCA) was conducted on the data matrix given by the m/z-representants. Principal component scores plots were created to visualize the separation between the pathogen groups. HCA with Euclidean distance measure and average linkage method [11] was used to analyze the hierarchical relationship between samples. Prior to PCA and HCA variables were standardized (mean centered and divided by the standard deviation).

To conduct supervised machine learning we selected the support vector machine algorithm (SVM) [12] with linear kernel and soft margin constant $C = 1$. Recall that the intuition behind the two-class SVM is to find a hyperplane $w^T x + b = 0$ with the largest separation between the two classes, where w is the so called weight vector, x a feature vector and b a scalar. Given n training samples $(x_i, y_i)_{i=1,\ldots,n}$, where x_i are feature vectors and $y_i \in \{-1, 1\}$ class labels, $w = (w_1, \ldots, w_n)$ and b result from the optimization problem of maximising the distance between all possible hyperplanes and closest (training) samples from each of the two classes. For the precise formulation of the underlying optimization problem we refer the reader to [12]. The class membership of any (test) feature vector x is then determined by $\text{sign}(w^T x + b) \in \{-1, 1\}$. A generalization of the binary SVM to a multi-class problem was suggested by Bottou et al. in [13]. Following [13], in case of $k \geq 3$ classes $c \in \{1, 2, \ldots, k\}$, k binary SVM classifiers are constructed by comparing each class against all remaining classes relabeled to a single new class, i.e. one-versus-all (OVA) model. The hyperplanes $w_c^T x + b_c$ are then used to determine the class membership of a feature vector x by $\text{argmax}_c(w_c^T x + b_c)$, i.e. the predicted class of a (test) feature x corresponds to the maximum value of k binary classifiers.

Applying this generalization of SVM to multi-class problems the, predictive power of the pathogen profiles was assessed in a leave-one-out cross-validation (LOOCV). In each of the 180 loops of the cross-validation one sample file was left out and assigned no label. The remaining 179 samples were used to generate pathogen profiles of m/z-representants and subsequently train a multi-class classifier with SVM by training 6 OVA models. The prediction was then made on the left out sample and compared with the original label. The overall accuracy was calculated as the ratio of correct predictions divided by 180. Note that all the processing steps needed to derive the m/z-representants data matrix of pathogen profiles were repeated in each loop of the cross-validation, see [14,15].

The internal mechanism of the SVM algorithm can also be used to rank the features according to their predictive power. One of the most popular methods is SVM Recursive-Feature-Elimination (SVM-RFE) which was originally proposed for two-class problems by Guyon et al. in [16]. It can be shown (see [16, section 2.5]), that the magnitude of

squared coefficients w_i^2 of the weight vector $w = (w_1, \ldots, w_n)$ can be used as a criterion for feature ranking. When removing a training feature x_i, the change in the cost function of the underlying optimization problem of SVM is approximately given by w_i^2. Therefore, the lower w_i^2 the less impact can be associated to the feature x_i when optimizing the separation of samples through hyperplanes. Using this, the ranking of the features is then done by eliminating recursively the features with the lowest impact [16, section 2.6]: the SVM classifier is trained on the complete data set and the feature with the lowest weight w_i^2 is removed giving it the lowest rank. A new SVM classifier is then trained on the remaining data set where again the feature with lowest weight is removed giving it the second lowest rank, etc. By repeating this process until all features are removed a ranking is produced for the complete feature set.

Applied to our data set with six pathogen labels, we defined six different two-class problems by comparing each pathogen against the other five labeled as a single new group. Subsequent application of SVM-RFE to each of the two-class problem gave six different rankings, one for each pathogen group. (The method of splitting the multi-class problem into several OVA two-class problems is one possible generalization of SVM-RFE to multi-class situation, see e.g. [17] and also [18, section 3.1]). For each of the six different rankings we selected top 10% m/z-representants per pathogen group. For later compound identification we treated each set separately by focusing on those m/z-representants with an elevated intensity for the pathogen group in question. That is, from each m/z-representant set we singled out only those representants for which the intensity was higher for the pathogen associated with m/z-representant set when compared to each of the other five. To achieve this, one-tailed Mann-Whitney U-test was applied in many-to-one fashion (comparing the pathogen with each of the five others) with the Hochberg adjustment [19] for p-values. The adjusted p-value threshold was set to 0.05 for the representant to be selected. Finally, the union over all selected m/z-representants was used for compound identification.

1.5. Putative identification workflow

The selective features were putatively assigned to chemical structures in a multi-step process, involving the freely available software SIRIUS [20–22] and a previously published MATLAB code [23], which was slightly modified i.e. debugged and adapted to process molecular formulas found from SIRIUS. The detailed parameters and settings of each step are described in the schematic below (see figure S2). In words, the individual steps can be summarized as follows:

1. Isotope filtering: The features were isotope filtered.
2. Checking for electrospray ionization (ESI) characteristic similarities: Features within one cluster were analyzed in respect to ESI characteristic similarities i.e. we checked for proton/alkali metal exchange, positive/negative ionization of the same compound or typical in-source collision induced dissociation (CID) neutral losses of common functional groups as outlined in [24]. Beside potential in-source H_2O adducts or losses, no repetitive similarity patterns were found.
3. Orbitrap reproducibility: The selective features from MS^1 TTOF 5600+ measurements were compared to the MS^1 detected features on the Orbitrap QE. Features, which were not detected (not reproducible) on the Orbitrap, were filtered out.
4. Orbitrap MS^2 interpretation: In-source CID of features within the same cluster were excluded by comparing a feature's MS^2 fragment peaks with the other features in the same cluster. Then, the MS^2 spectra were analyzed with SIRIUS (4.4.29) [20–22]. The reported structures correspond to the top SIRIUS hit which is reported in the Kyoto Encyclopedia of Genes and Genomes (KEGG) database or the Human Metabolome Database (HMDB). Molecular formulas, which could not be identified by MS^2 spectra (due to low abundance, low molecular weight, interference), were fed into the MATLAB code FindCommonKeggPathways.m from [23] to search for a KEGG pathway linking the molecular formulas with each another. These Molecular

formulas within one cluster being metabolically linked according to KEGG are also reported.

5. In a literature search, we checked whether the putatively identified compounds were reported in the context of bacteria volatiles.
6. Finally, we checked the plausibility of the putatively identified compounds, i.e. we checked if their volatility is sufficient to be detectable with SESI, and excluded compounds with very low SIRIUS MS^2 scores, i.e. lower than -300.

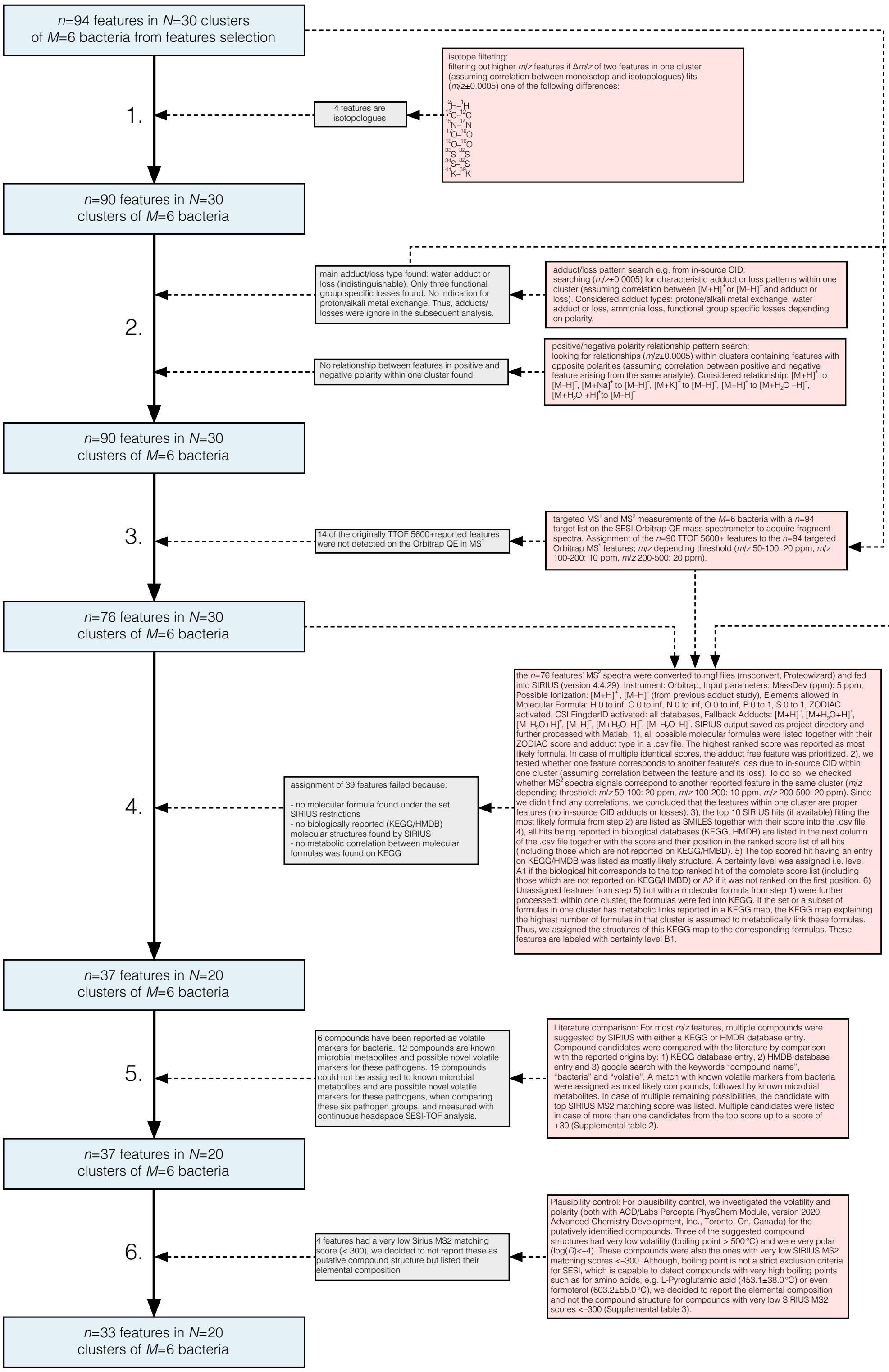

Figure S2. Process schema of putative assignment

2. Tables associated with putative compound identification

- Table S2 (link): Target list with structure.
- Table S3 (link): Putative markers literature comparison.
- Table S4 (link): Plausibility control.
- Table S5 (link): List of putatively identified compounds.

Pediatric exhalomics group (PEG)

Astghik Baghdasaryan, Christoph Berger, Christian Bieli, Tobias Bruderer, Naemi Haas, Martin Hersberger, Katharina Heschl, Demet Inci, Andreas Jung, Malcolm Kohler, Srdjan Micic, Alexander Moeller, Simona Müller, Nathan Perkins, Renate Spinas, Bettina Streckenbach, Jakob Usemann, Ronja Weber and Renato Zenobi.

References

1. Kessner, D.; Chambers, M.; Burke, R.; Agus, D.; Mallick, P. ProteoWizard: open source software for rapid proteomics tools development. *Bioinformatics (Oxford, England)* **2008**, *24*, 2534–2536. 18606607[pmid], doi:10.1093/bioinformatics/btn323.
2. Fritsch, F.N.; Carlson, R.E. Monotone Piecewise Cubic Interpolation. *SIAM Journal on Numerical Analysis* **1980**, *17*, 238–246.
3. Rees, C.A.; Burklund, A.; Stefanuto, P.H.; Schwartzman, J.D.; Hill, J.E. Comprehensive volatile metabolic fingerprinting of bacterial and fungal pathogen groups. *Journal of Breath Research* **2018**, *12*, 026001. doi:10.1088/1752-7163/aa8f7f.
4. Weber, R.; Haas, N.; Baghdasaryan, A.; Bruderer, T.; Inci, D.; Micic, S.; Perkins, N.; Spinas, R.; Zenobi, R.; Moeller, A. Volatile organic compound breath signatures of children with cystic fibrosis by real-time SESI-HRMS. *ERJ Open Research* **2020**, *6*.
5. Nowak, N.; Engler, A.; Thiel, S.; Stöberl, A.S.; Sinues, P.; Zenobi, R.; Kohler, M. Validation of breath biomarkers for obstructive sleep apnea. *medRxiv* **2021**. doi:10.1101/2021.03.16.21253612.
6. Gaisl, T.; Bregy, L.; Stebler, N.; Gaugg, M.T.; Bruderer, T.; García-Gómez, D.; Moeller, A.; Singer, F.; Schwarz, E.I.; Benden, C.; others. Real-time exhaled breath analysis in patients with cystic fibrosis and controls. *Journal of breath research* **2018**, *12*, 036013.
7. Sinues, P.M.L.; Landoni, E.; Miceli, R.; Dibari, V.F.; Dugo, M.; Agresti, R.; Tagliabue, E.; Cristoni, S.; Orlandi, R. Secondary electrospray ionization-mass spectrometry and a novel statistical bioinformatic approach identifies a cancer-related profile in exhaled breath of breast cancer patients: a pilot study **2015**. *9*, 031001. doi:10.1088/1752-7155/9/3/031001.
8. Mann, H.B.; Whitney, D.R. On a Test of Whether one of Two Random Variables is Stochastically Larger than the Other. *Ann. Math. Statist.* **1947**, *18*, 50–60. doi:10.1214/aoms/1177730491.
9. Benjamini, Y.; Hochberg, Y. Controlling The False Discovery Rate - A Practical And Powerful Approach To Multiple Testing. *J. Royal Statist. Soc., Series B* **1995**, *57*, 289 – 300. doi:10.2307/2346101.
10. Shapiro, S.S.; Wilk, M.B. An analysis of variance test for normality (complete samples)†. *Biometrika* **1965**, *52*, 591–611. doi:10.1093/biomet/52.3-4.591.
11. Sokal, R.; Michener, C. A Statistical Method of Evaluating Systematic Relationships. *The University of Kansas Science Bulletin* **1958**, *38*, 1409–1438.
12. Cortes, C.; Vapnik, V. Support-vector networks. *Machine Learning* **1995**, *20*, 273–297. doi:10.1007/BF00994018.
13. Bottou, L.; Cortes, C.; Denker, J.S.; Drucker, H.; Guyon, I.; Jackel, L.D.; LeCun, Y.; Muller, U.A.; Sackinger, E.; Simard, P.; Vapnik, V. Comparison of classifier methods: a case study in handwritten digit recognition. Proceedings of the 12th IAPR International Conference on Pattern Recognition, Vol. 3 - Conference C: Signal Processing (Cat. No.94CH3440-5), 1994, Vol. 2, pp. 77–82 vol.2. doi:10.1109/ICPR.1994.576879.
14. Varma, S.; Simon, R. Bias in error estimation when using cross-validation for model selection. *BMC Bioinformatics* **2006**, *7*, 91. doi:10.1186/1471-2105-7-91.
15. Bermingham, M.L.; Pong-Wong, R.; Spiliopoulou, A.; Hayward, C.; Rudan, I.; Campbell, H.; Wright, A.F.; Wilson, J.F.; Agakov, F.; Navarro, P.; Haley, C.S. Application of high-dimensional feature selection: evaluation for genomic prediction in man. *Scientific Reports* **2015**, *5*, 10312. doi:10.1038/srep10312.
16. Guyon, I.; Weston, J.; Barnhill, S.; Vapnik, V. Gene Selection for Cancer Classification using Support Vector Machines. *Machine Learning* **2002**, *46*, 389–422. doi:10.1023/A:1012487302797.
17. Ramaswamy, S.; Tamayo, P.; Rifkin, R.; Mukherjee, S.; Yeang, C.H.; Angelo, M.; Ladd, C.; Reich, M.; Latulippe, E.; Mesirov, J.P.; Poggio, T.; Gerald, W.; Loda, M.; Lander, E.S.; Golub, T.R. Multiclass cancer diagnosis using tumor gene expression signatures. *Proceedings of the National Academy of Sciences* **2001**, *98*, 15149–15154. doi:10.1073/pnas.211566398.
18. Zhou, X.; Tuck, D.P. MSVM-RFE: extensions of SVM-RFE for multiclass gene selection on DNA microarray data. *Bioinformatics* **2007**, *23*, 1106–1114. doi:10.1093/bioinformatics/btm036.
19. HOCHBERG, Y. A sharper Bonferroni procedure for multiple tests of significance. *Biometrika* **1988**, *75*, 800–802. doi:10.1093/biomet/75.4.800.
20. Dührkop, K.; Shen, H.; Meusel, M.; Rousu, J.; Böcker, S. Searching molecular structure databases with tandem mass spectra using CSI:FingerID. *Proceedings of the National Academy of Sciences* **2015**, *112*, 12580–12585. doi:10.1073/pnas.1509788112.
21. Kai Dührkop et al. "SIRIUS 4: a rapid tool for turning tandem mass spectra into metabolite structure information". In: Nature Methods 16.4 (Apr. 2019), pp. 299–302. issn: 1548-7105. doi: 10.1038/s41592-019-0344-8.

22. Ludwig, M.; Nothias, L.F.; Dührkop, K.; Koester, I.; Fleischauer, M.; Hoffmann, M.A.; Petras, D.; Vargas, F.; Morsy, M.; Aluwihare, L.; Dorrestein, P.C.; Böcker, S. ZODIAC: database-independent molecular formula annotation using Gibbs sampling reveals unknown small molecules. *bioRxiv* **2019**. doi:10.1101/842740.
23. Gaugg, M.T. HRMS Breath Analysis Matlab Toolbox, 2019. doi:10.3929/ethz-b-000335190.
24. Niessen, W.M.; others. *Interpretation of MS-MS mass spectra of drugs and pesticides*; John Wiley & Sons, 2017.
