# Supplementary figures and images for: Differentiation of Cystic Fibrosis-Related Pathogens by Volatile Organic Compound Analysis with Secondary Electrospray Ionization Mass Spectrometry
Source: Metabolites. 2021 Nov 11;11(11):773. doi: 10.3390/metabo11110773 (PMC8617967; doi:10.3390/metabo11110773)

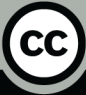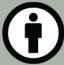

BY

Supplement: Supplementary file 1 [file metabolites-11-00773-s001.zip › metabolites-1444134-supplementary -revise/Definitions/logo-ccby-eps-converted-to.pdf]

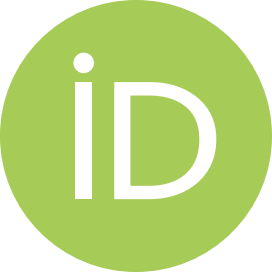

Supplement: Supplementary file 1 [file metabolites-11-00773-s001.zip › metabolites-1444134-supplementary -revise/Definitions/logo-orcid-eps-converted-to.pdf]

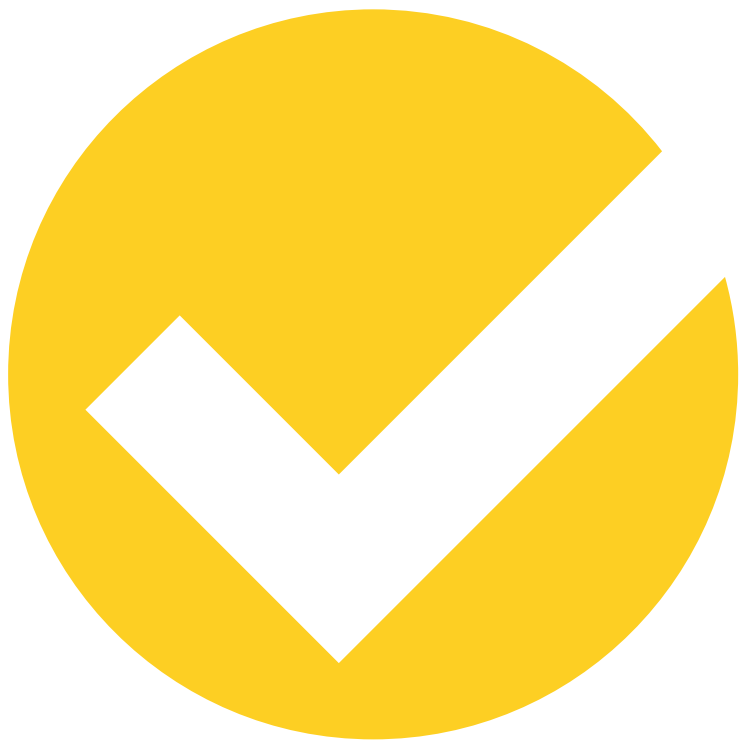

check for  
updates

Supplement: Supplementary file 1 [file metabolites-11-00773-s001.zip › metabolites-1444134-supplementary -revise/Definitions/logo-updates.pdf]

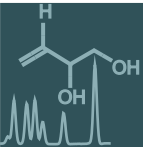

*metabolites*

Supplement: Supplementary file 1 [file metabolites-11-00773-s001.zip › metabolites-1444134-supplementary -revise/Definitions/metabolites-logo-eps-converted-to.pdf]

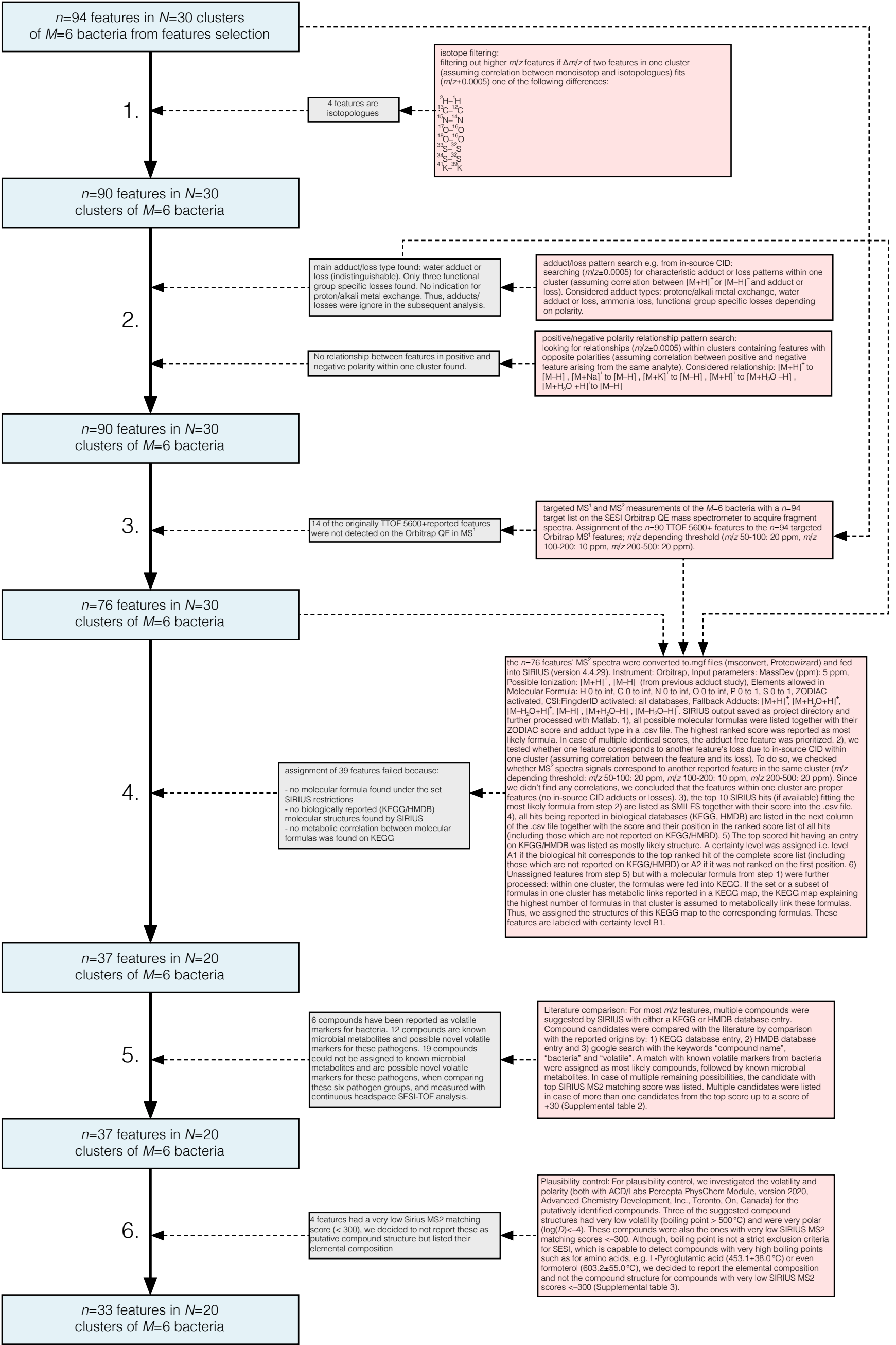

Supplement: Supplementary file 1 [file metabolites-11-00773-s001.zip › metabolites-1444134-supplementary -revise/Figures/compound_identification_workflow.pdf]

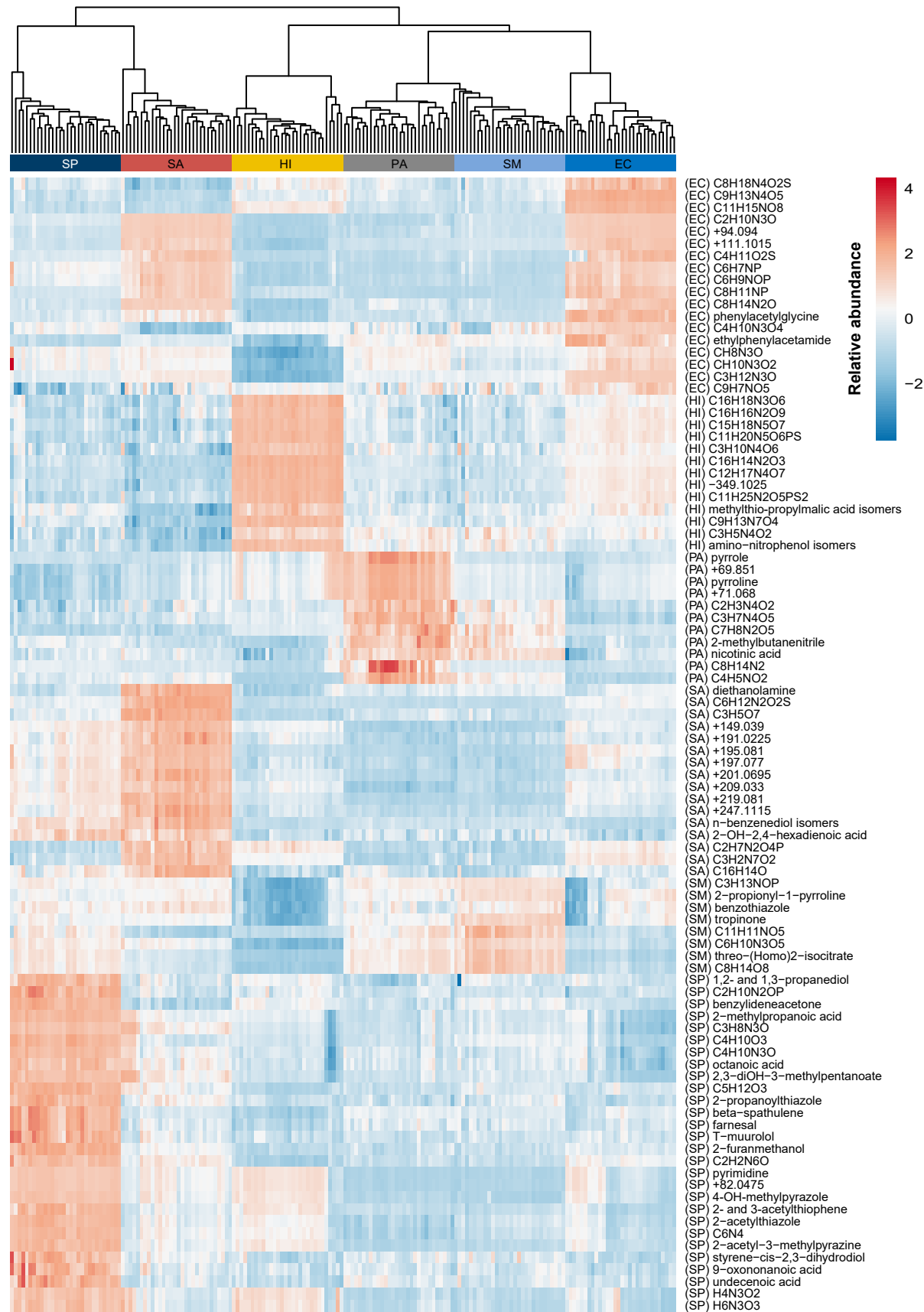

Supplement: Supplementary file 1 [file metabolites-11-00773-s001.zip › metabolites-1444134-supplementary -revise/Figures/heatmap_with_compound_names.pdf]

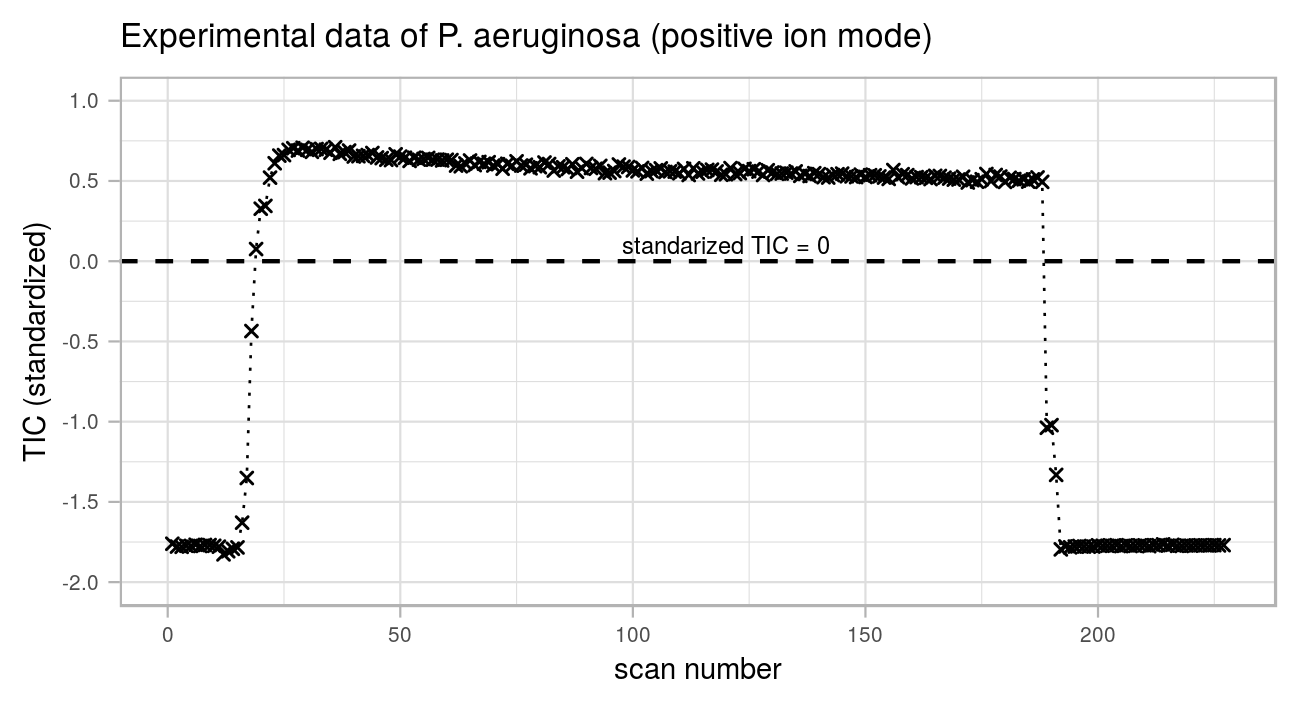

Supplement: Supplementary file 1 [file metabolites-11-00773-s001.zip › metabolites-1444134-supplementary -revise/Figures/tic_example.png]
