# Supplementary material for: Differentiation of Cystic Fibrosis-Related Pathogens by Volatile Organic Compound Analysis with Secondary Electrospray Ionization Mass Spectrometry
Source: Metabolites. 2021 Nov 11;11(11):773. doi: 10.3390/metabo11110773 (PMC8617967; doi:10.3390/metabo11110773)
Supplement: Supplementary file 1 [file spectra_overview.pdf › metabolites-1444134-supplementary -revise/Figures/spectra_overview.pdf]

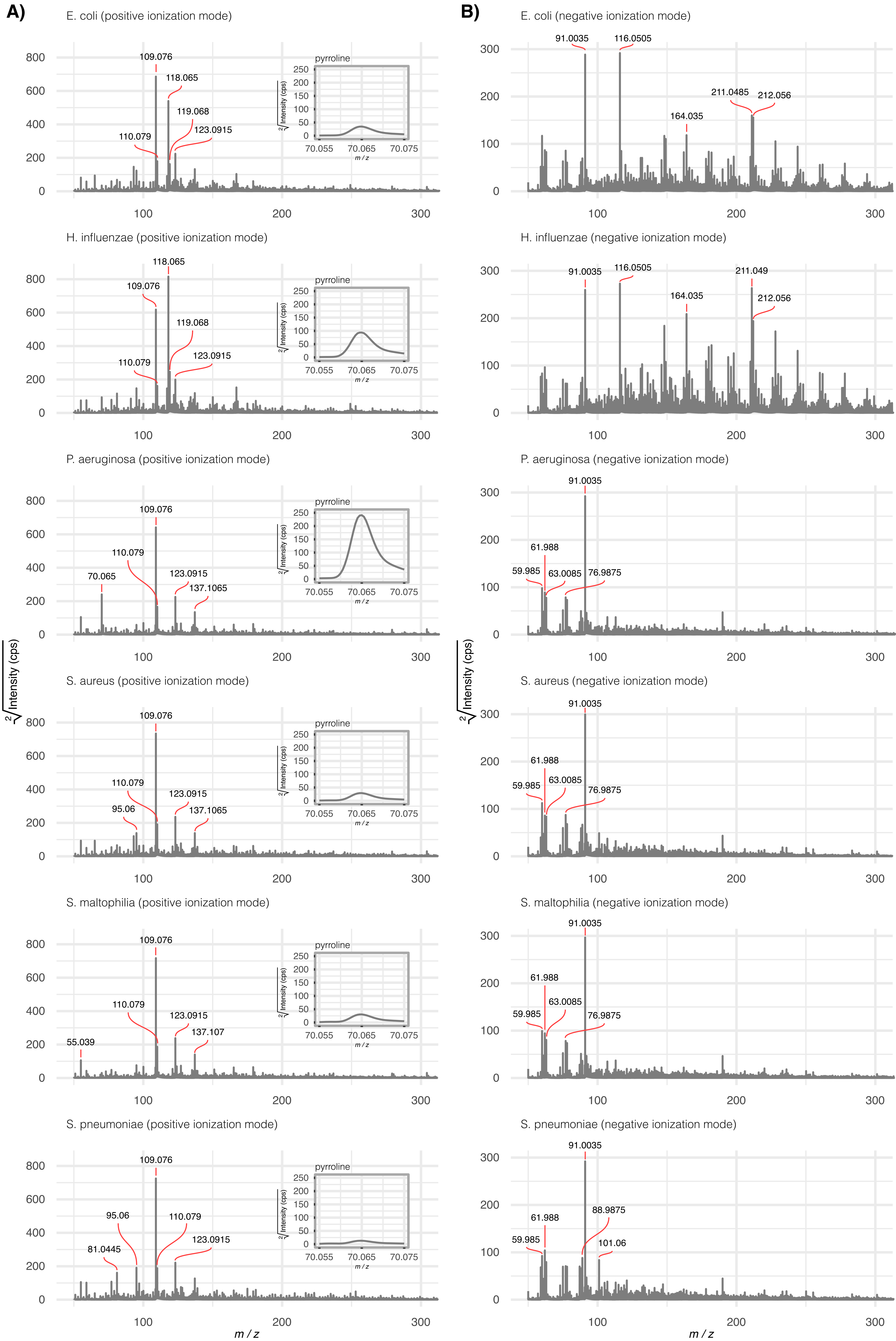
A)
B)
E. coli (positive ionization mode)
E. coli (negative ionization mode)
H. influenzae (positive ionization mode)
H. influenzae (negative ionization mode)
P. aeruginosa (positive ionization mode)
P. aeruginosa (negative ionization mode)
S. aureus (positive ionization mode)
S. aureus (negative ionization mode)
S. maltophilia (positive ionization mode)
S. maltophilia (negative ionization mode)
S. pneumoniae (positive ionization mode)
S. pneumoniae (negative ionization mode)
pyrroline
Intensity (cps)
m / z
109.076
118.065
119.068
110.079
123.0915
137.1065
137.107
70.065
95.06
55.039
81.0445
91.0035
116.0505
211.0485
211.049
212.056
164.035
61.988
59.985
63.0085
76.9875
88.9875
101.06
